# Rubber Fatigue Revisited: A State-of-the-Art Review Expanding on Prior Works by Tee, Mars and Fatemi

**DOI:** 10.3390/polym17070918

**Published:** 2025-03-28

**Authors:** Xiaoli Wang, Ramin Sedaghati, Subhash Rakheja, Wenbin Shangguan

**Affiliations:** 1 School of Automobile and Transportation Engineering, Guangdong Polytechnic Normal University, Guangzhou 510665, China; 2 Department of Mechanical, Industrial and Aerospace, Concordia University, Montreal, QC H3G 1M8, Canada; 3 School of Mechanical and Automotive Engineering, South China University of Technology, Guangzhou 510641, China; sgwb@163.com

**Keywords:** rubber and elastomeric material, fatigue, life prediction, experiment, magnetorheological elastomers

## Abstract

Rubber materials can endure substantial deformation while avoiding permanent damage or rupture, making them highly suitable for applications in the automotive industry and other sectors, particularly for noise and vibration reduction. However, rubber experiences degradation over time as defects or cracks appear and propagate under fluctuating loads. Therefore, it is of critical importance to prevent the failure of rubber components during service. As highlighted in prior literature surveys by Tee et al. in 2018, Mars and Fatemi in 2002 and 2004, significant research has focused on the mechanics and analysis of rubber fatigue. This body of work has grown rapidly and continues to evolve. Therefore, this study aims to compile and analyze the vast body of recent research on rubber fatigue conducted over the last decade, supplementing the reviews by Tee et al. in 2018, Mars and Fatemi in 2002 and 2004. The gathered studies were analyzed to identify current trends and emerging research gaps in the fatigue study of rubber, including advanced composite rubber materials such as magnetorheological elastomers (MREs). This review emphasizes the analysis techniques and fatigue experiments available for fatigue life prediction in rubber materials, while illustrating their practical applications in engineering analyses through specific examples.

## 1. Introduction

Rubber’s exceptional properties—such as high elastic recovery, flexibility, extensibility, low cost, and light weight—make it invaluable across various industries. In the automotive sector, it is widely used in applications like engine mounts, tires, and vibration isolators, among other things. Advanced rubber materials, such as magnetorheological elastomers (MREs), are being applied in cutting-edge fields like soft robotics, electronic skins, and health monitoring systems due to their ability to significantly alter their properties in response to external stimuli. Furthermore, their potential integration with carbon nanotubes or conjugated polymers to develop stretchable conductors or semiconductors positions them as an ideal choice for future technologies, including self-actuators, epidermal electronics, and implantable sensors [1,2].

In service, most rubber components are subjected to fluctuating loads, making them susceptible to fatigue failure. In light of the essential need to prevent such failures, research on rubber fatigue has grown significantly over the past few decades. As noted in Ref. [3], the fatigue behavior of elastomers is influenced by four primary factors: the history of mechanical loading, environmental exposure, rubber composition, and the energy dissipation properties inherent in the material’s constitutive behavior. Among these factors, mechanical fatigue—particularly the initiation and growth of cracks within the rubber—frequently plays a critical role in determining long-term durability, which is the focus of this study. It should be noted that environmental conditions such as temperature, UV exposure, oxygen, and chemical exposure are also critical for long-term rubber fatigue. Readers can refer to Refs. [4,5] for further details.

**Figure 1:**
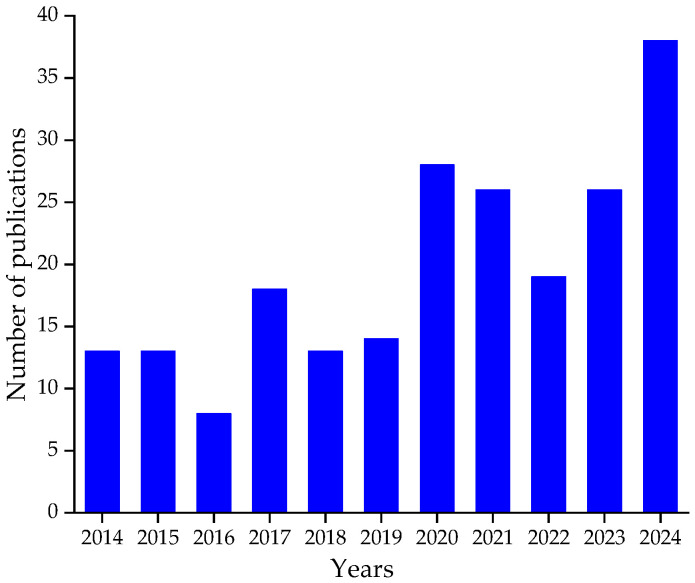
Quantity of relevant literature publications over the past decade.

Many methods for the physical testing of rubber are based on techniques originally developed for metals. However, these procedures frequently prove less effective for elastomers owing to the intricate interactions among polymers, fillers, plasticizers, and additional additives [6,7]. The challenges in estimating the fatigue life of rubber-like materials stem from factors such as crystallization, heterogeneous formulations, and complex mechanical behavior. Since Mars and Fatemi’s comprehensive literature review in 2002 [8], approximately 400 papers have been published on the subject, with around 200 related to rubber fatigue modeling and experiments in the past decade, as shown in Figure 1. The data shown in this figure were gathered from three bibliographic databases: Google Scholar, Engineering Village, and Web of Science, along with the Sofia Discovery tool at Concordia University, using keywords like fatigue, life, rubber, and predictor. The findings underscore the pressing need for a better understanding and prediction of fatigue failure in rubber materials. Motivated by this, the current work aims to analyze the extensive body of research on rubber fatigue since the reviews by Tee et al. [9] and Mars and Fatemi [3,8]. The objective is to update these reviews to incorporate recent and previously overlooked developments, although some of the information presented here has been discussed in earlier works [1,9,10].

**Figure 2:**
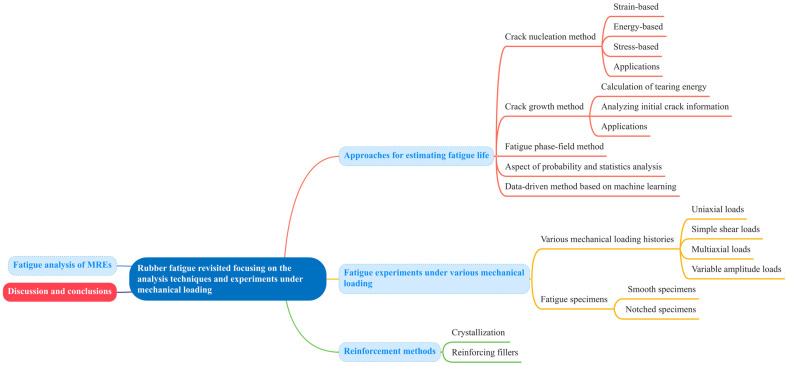
A graphical illustration of the work.

This paper is structured as follows (see Figure 2): Section 2 reviews various analytical approaches for rubber, rubber-like materials, and elastomers, along with their applications in different rubber components. Section 3 explores fatigue experiments reported in the literature, focusing on two key aspects: types of mechanical loading and fatigue specimen selection. Section 4 discusses reinforcement methods aimed at enhancing the fatigue resistance of rubber materials. In particular, recent advancements in the fatigue performance of magnetorheological elastomers (MREs) are presented in Section 5. Lastly, Section 6 outlines future trends in this field and concludes the paper.

## 2. Modeling of Fatigue Life for Rubber-like Materials

In general, rubber fatigue involves a period during which cracks appear due to fluctuating loading in regions previously devoid of detectable defects, succeeded by a subsequent phase where these initiated cracks propagate until failure occurs. In this vein, two classic approaches in the fatigue analysis of rubber are available: the crack nucleation (or initiation) method and the crack growth (or propagation) method. New approaches, such as the fatigue phase-field method, the probabilistic fatigue analysis method, and the data-driven method based on machine learning, have emerged in recent years as well. This section presents five different approaches for rubber fatigue analysis and some application examples.

### 2.1. Crack Nucleation Method

The crack nucleation approach is based on continuum mechanics [11], damage mechanics [12], and configuration mechanics [13], where the material is typically assumed to maintain internal homogeneity and continuity from the onset of loading until the point of crack initiation. The crack nucleation approach considers that a material possesses an inherent lifespan dictated by the stress or strain history at each material point in the body, regardless of the specimen geometry or the type of loading. A damage parameter (also known as fatigue predictor, criteria, prediction factors, mechanical parameters, damage indicators, etc.), which correlates with the local force, deformation, or energy state of the material, is of crucial importance to be determined in advance. The damage parameter can typically be expressed in terms of stresses and strains, concepts well-known to designers, which makes the approach relatively convenient and popular in engineering applications. In particular, the approach is especially suitable for scenarios where the initial defects, which ultimately determine component life, are significantly smaller than the component’s features and where analyzing the spatial distribution of fatigue life is essential.

The crack nucleation approach was initially adapted for rubbers by Cadwell et al. [14] and remains in use today [15]. In the crack nucleation approach, the end of life is empirically determined by the number of cycles to cause a reduction in mechanical stiffness (or load drop), the emergence of a crack of a specified dimension, or complete rupture [10,11]. The end of life of a material is considered to have a power relationship with its damage parameter.

The most commonly used damage parameters can be categorized into strain-based, energy-based, and stress-based damage parameters. Moreover, when using damage parameters to characterize fatigue lives, a choice has to be made to use either the maximum value of a parameter or the range for a parameter during one load cycle. This is an important issue since the values can differ significantly for certain load paths [16].

The determination of the multiaxial fatigue damage parameter over a single cycle is frequently accomplished by utilizing cycle-defined quantities such as stress, strain, or energy amplitudes, mean values, and peak values, which are often directly applied to the rubbers. However, in reality, such calculations are not suggested due to the nonlinear behavior linked to finite strains and the near-incompressibility characteristic of rubber materials. Thus, it is advisable to calculate the amplitude or peak value from the time history of the multiaxial fatigue damage parameter of interest [17]. Furthermore, certain existing fatigue life models [16] are based on a certain combination of stress, strain, and strain energy, depending on the type of material studied.

#### 2.1.1. Strain-Based Damage Parameters

Strain serves as a convenient damage parameter in cases involving displacement control, because it can be readily determined from the imposed displacement and does not require the use of a constitutive equation to predict strain levels during the analysis process. Furthermore, experimental observations in rubbers show that the cracks frequently nucleate on a plane perpendicular to the direction of maximum principal strain.

All strains applied to a small volume element can be simplified into three mutually perpendicular extensions acting on the element, called the principal strains where the maximum one is known as the first or maximum principal strain. The strain-based damage parameters used in rubber fatigue encompass the maximum principal strain, octahedral shear strain, peak shear strain, and various effective or equivalent strain measures.

As for the maximum principal strain, five formulations of strain are used as damage parameters considering the nonlinear and finite deformation characteristics of rubber-like materials. The published maximum principal strains [18] consist of the stretch ratio (elongation), engineering strain, Green–Lagrange strain, Almansi–Euler strain, and logarithmic strain (for simplicity, strain limits are confined to amplitudes and maximum values herein and are omitted in the description of damage parameters, unless specified otherwise). Theoretically, all of the strain measures could serve as damage parameters for estimating the fatigue life since they can be converted into each other.

The stretch ratio (elongation) λ is defined as the quotient of the deformed length *l* and the undeformed length *l*_0_ of a specimen:

(1) $$\lambda =\frac{l}{l_{0}}$$

The engineering strain $\varepsilon_{E}$, the Green–Lagrange strain $\varepsilon_{G-L}$, the Almansi–Euler strain $\varepsilon_{A-E}$, and the logarithmic strain $\varepsilon_{L}$, are, respectively, obtained from the stretch ratio λ as follows:

(2) $$\varepsilon_{E}=\lambda -1$$

(3) $$\varepsilon_{G-L}=\frac{\lambda^{2}-1}{2}$$

(4) $$\varepsilon_{A-E}=\frac{\lambda^{2}-1}{2\lambda^{2}}$$

(5) $$\varepsilon_{L}=\int_{l_{0}}^{l}\frac{dl}{l}=\ln\left(\frac{l}{l_{0}}\right)=\ln(\lambda )$$

and the octahedral shear strain $\varepsilon_{o}$, which is also taken as a damage parameter in rubber fatigue [17,18], can be described as follows:

(6) $$\begin{array}{l} \varepsilon_{o}=\frac{1}{3}\sqrt{{\left(E_{11}-E_{22}\right)}^{2}+{\left(E_{22}-E_{33}\right)}^{2}+{\left(E_{33}-E_{11}\right)}^{2}+6(E_{12}^{2}+E_{23}^{2}+E_{31}^{2})}\\ =\frac{\sqrt{2}}{6}\left|\lambda^{2}-\frac{1}{\lambda }\right| \end{array}$$

In which $E_{ij}\;(i,j=1,2,3)$ are the components of the Green–Lagrange strain tensor E, respectively.

According to the finite deformation theory, Green–Lagrange strain and Almansi–Euler strain accurately capture the nonlinear deformation and large strain characteristics of elastomeric materials. However, the engineering strain (also called nominal strain), a more obvious choice that was often used as a damage parameter in previous decades, is easy to calculate using the definition and the imposed displacement during experiments. The earliest fatigue study in rubber [14] utilized engineering strain limits (e.g., minimum strain and strain range) as a damage parameter to represent the load imposed on the rubber specimens. The same approach (based simply on minimum engineering elongations and amplitudes) was applied in order to study the effect of initial elongations on four types of synthetic rubbers [19].

However, when correlated with the maximum principal stretch (or strain), fatigue life was observed to be longer under simple tension than under equi-biaxial tension [20,21]. The difference was more pronounced in natural rubber (NR) and less evident in styrene butadiene rubber (SBR). Subsequently, Ro [22] re-examined the data from these studies, employing the maximum principal strain, the maximum shearing strain, total strain energy, and distortional strain energy as damage parameters. It was concluded that satisfactory correlations could be attained using the maximum principal strain at higher strain levels (>250%) for SBR materials, whereas the maximum shearing strain with the linear model, and particularly the total strain energy, performed more effectively at relatively lower strains (<100%). It was also found that the distortional strain energy predicted larger values of fatigue life than the total strain energy.

On the other hand, Zine et al. [23] discovered that both the maximum principal Green–Lagrange strain and the strain energy density (SED) serve as effective damage indicators for predicting rubber fatigue life, whereas the maximum principal engineering strain fails to adequately fit both uniaxial and biaxial (pure shear) experimental results.

Detailed overviews of the different predictors employed in such analyses can be found in reviews [1,9,10], but the number of studies using strain-based life approaches continues to increase. Various strain-based damage parameters are increasingly used in rubber nucleation life prediction in the literature, prompting researchers to assess their effectiveness across different types of rubber.

Shangguan et al. [18] and Belkhiria et al. [24] successively utilized the maximum principal logarithmic strain, engineering strain, Green–Lagrange strain, Almansi–Euler strain, and octahedral shear strain, applying these measures to predict the uniaxial fatigue life of carbon-filled NR and SBR, respectively, and subsequently validated the accuracies through uniaxial tensile fatigue experiments. Both found that the above-mentioned damage parameters have excellent fatigue–life estimations. However, the former concluded that the correlation between the maximum principal Green–Lagrange strain and the experimental results had the best performance, whereas the latter suggested that the model based on the maximum octahedral shear strain was the most precise. These contrasting results are potentially attributable to the fatigue behaviors differences between filled NR and SBR.

Very recently, Luo et al. [25] performed fatigue failure tests utilizing hourglass-shaped rubber specimens and empirically obtained the conventional S–N curve by employing the maximum principal strain (it is possibly they used the Green–Lagrange strain; there was no specified description of the strain type in their work) as the fatigue parameter to establish the correlation between fatigue life and temperature. Recent research indicates a growing preference for the maximum principal Green–Lagrange strain as a damage parameter for rubber fatigue within the industry [26,27]. However, it is important to note that the aforementioned studies primarily focus on uniaxial or simple fatigue loadings for rubber-like materials.

To explore multiaxial fatigue damage parameters in rubber materials, Mars and Fatemi [17] investigated the maximum principal engineering strain (or stretch), SED, and octahedral shear strain, as well as the proposed plane-dependent cracking energy density (CED)—discussed in a later section. They suggested that, of the three scalar equivalence criteria evaluated, the maximum principal strain demonstrated superior significance and reliability. Their findings revealed that this particular criterion generally exhibited the strongest correlation with the CED parameter, whereas the SED displayed the most substantial deviation from the observed data.

Recently, Ayoub et al. [12] further investigated the multiaxial fatigue prediction capabilities of several damage parameters, including the maximum principal stretch, maximum principal Cauchy stress, and SED, as well as critical plane parameters like CED, developed by Mars and Fatemi [17], an equivalent stress predictor proposed by Ayoub et al. [12], and a configurational stress predictor outlined by Verron and Andriyana [13]. Through combined tension–compression and torsion fatigue tests on SBR material, they found significant limitations in utilizing maximum principal stretch as a damage parameter for fatigue life prediction across both uniaxial and multiaxial loading conditions. The research outcomes demonstrated that alternative mechanical parameters, including maximum principal Cauchy stress, the stress-based predictive model developed by Ayoub et al. [12], and the CED parameter, exhibited superior capability as effective damage indicators for accurate multiaxial fatigue life estimation. They also recommended that the predictors, which performed well in estimating the fatigue life of the studied SBR material, should be further tested using more complex methods, such as non-proportional fatigue tests. Additionally, they noted that current fatigue life predictors do not consider the impact of self-heating on fatigue life, and research into this area could be highly valuable.

Very recently, Luo [28,29,30] proposed the effective tensile strain criterion, shown in Equation (7), to model multiaxial fatigue and evaluated its capability in different loading conditions utilizing published experimental data [31] for applications such as suspension components.

(7) $$\varepsilon_{f}=\sqrt{\varepsilon_{f1}^{2}+A_{1}\varepsilon_{f2}^{2}+A_{2}\varepsilon_{f3}^{2}},\;\varepsilon_{f1}\geq \varepsilon_{f2}\geq \varepsilon_{f3}$$

In Equation (7), *A*_1_ and *A*_2_ are weighting functions; $\varepsilon_{fi}(i=1,2,3)$ are the principal components of $\varepsilon_{f}$, respectively.

They also pointed out that additional research may be necessary to explore other potential conditions and to compare the results with existing published data in order to further validate this approach.

#### 2.1.2. Energy-Based Damage Parameters

SED was adopted as a damage parameter for forecasting fatigue crack nucleation with the significant advancement of fracture mechanics in rubber material science [32]. It was computed using the experimental axial load and torque by numerically integrating the experimental stress–strain results [16]. In the early research era, Ro [22] demonstrated the comparative effectiveness of SED as a damage parameter for fatigue life assessment in SBR. Their experimental results revealed that SED outperformed alternative strain-based damage indicators under non-relaxing uniaxial loading conditions. Similarly, Abraham et al. [6] pointed out that SED serves as a more reliable indicator for fatigue life prediction in filled SBR and EPDM (ethylene propylene diene monomer) rubber compounds subjected to uniaxial tensile and compressive loading conditions. Their experimental findings provided substantial evidence that energy-based parameters, rather than conventional stress or strain measures, play a predominant role in determining the fatigue characteristics of these elastomeric materials. Shangguan et al. [18] utilized the SED computed from the loading and unloading stress–strain curves to the predict uniaxial fatigue of filled NR. Zine et al. [23] implemented SED as a damage parameter for fatigue life assessment uniaxial tension and pure shear fluctuating loading and found that SED seemed like a reliable damage indicator.

However, Ayoub et al. [12] clearly showed that the SED could not be taken as a fatigue indicator for filled SBR, since it could not unify the experimental data corresponding to tension and torsion tests. Findley et al. [33] found via experimentation that specimen failure was still observed to occur under constant SED but with varying stresses for metals, which indicated that the SED is not valid for describing the mechanism of fatigue for combined stress. Similarly, Moon et al. [34] utilized the maximum principal strain and SED obtained from finite element analysis (FEA) to characterize the lifetime of 3D dumbbell specimens under loadings of varying mean displacements and amplitude displacements and used the model for the fatigue prediction of a suspension bush. They determined that, likely due to shear deformation, the predicted lifetime of the suspension bush is underestimated, with SED showing a significant discrepancy of 77%, and the maximum principal strain displaying a discrepancy of 52%. They suggested that normal strain and shear strain should be included for better accuracy, but did not provide any details.

The conditions under which the SED may be taken as an effective damage parameter are limited, and under such conditions the SED is uniquely related to the energy release rate (described in Section 2.2) to some extent. For such a correlation to be established, several fundamental assumptions are typically incorporated: (1) the propagation of cracks maintains self-similar characteristics throughout the growth process, (2) the strain gradient in the far-field region exhibits negligible influence on crack behavior, and (3) the material experiences a uniaxial tensile stress state without complex stress interactions. For instance, SED is a proper damage parameter under uniaxial relaxing tension and pure shear loading since SED is equal to CED under such simple loading conditions [23,35].

Indeed, Poisson et al. [36] conducted a comparative experimental analysis of the fatigue behavior of polychloroprene rubber (CR) under both uniaxial non-relaxing and biaxial loading conditions. They revealed significant limitations in utilizing first principal stress and strain energy density (SED) as reliable predictors for fatigue life estimation. Instead, their research findings suggested that dissipated energy seems to be the most relevant criteria irrespective of the loading paths. Marco et al. [37] also established a correlation between fatigue lifetime and energy dissipation for natural rubber (NR) compounds by integrating the evolution of dissipated energy and the quantitative assessment of crack surface density distribution.

In theory, when applied as a scalar criterion, neither the SED, dissipated energy, nor maximum principal strain can predict the specific orientation of the crack surface. As a result, they fail to account for the fact that, under multiaxial conditions, only a portion of the total energy spent contributes to the crack nucleation process. Additionally, these criteria can remain constant and predict infinite life, especially in non-proportional loading scenarios, even though they actually lead to finite life (due to cyclic opening and shearing of embedded flaws with a constant criterion value). Lastly, these three parameters do not consider crack closure and fail to predict significant differences in life between simple tension and simple compression loadings.

To solve the issue mentioned, Mars [35] proposed a novel predictor termed cracking energy density (CED), operating as a critical plane-specific indicator, which quantitatively characterizes the fraction of strain energy density (SED) that becomes available for release through crack propagation along specific material planes. Using the CED parameter, one can calculate the energy release rate (tearing energy) for an arbitrarily complex strain history and determine the crack orientation where the maximum CED is reached. Mars and Fatemi [11] demonstrate, based on physical reasoning and robustness, that CED can unify multiaxial fatigue data. However, further investigation is needed to assess its applicability to elastomeric materials beyond filled NR.

Accordingly, Harbour et al. [16,38] successfully implemented this methodological framework to evaluate fatigue life and crack orientation characteristics in both NR and SBR materials under complex loading scenarios, including variable amplitude and multiaxial loading conditions. As for fatigue life prediction, they found that CED produced the best correlation of the constant amplitude fatigue life data for both NR and SBR materials, while the NR results tended to have better correlations than the SBR results. Regarding crack orientation prediction, they demonstrated that both the CED approach and normal strain approach exhibited satisfactory predictive capabilities for specific loading conditions, particularly in axial and multilevel axial testing scenarios in each material. However, these approaches demonstrated significant limitations when applied to more complex loading patterns, notably in fully reversed torsional loading conditions, where prediction accuracy substantially decreased. They proposed an innovative fatigue life prediction methodology that performed critical plane identification through maximum normal strain approach and subsequent fatigue life estimation using CED calculations on the identified plane. This dual-approach technique offers significant computational advantages, since the maximum normal strain remains regardless of constitutive behavior for a given deformation, thereby enabling efficient multi-plane analysis without requiring material-specific constitutive modeling. Zine et al. [39] further confirmed the enhanced predictive capability of the CED approach when compared to conventional SED methodology in the fatigue analysis. The CED remains in frequent use and has been verified for different loadings and applications [23,40,41,42,43].

In a recent study, Pan et al. [44] performed a comparative assessment of five prominent fatigue life prediction methodologies using experimental data obtained from filled vulcanized NR specimens subjected to multiaxial loading conditions (involving axial-shear combined strains under three distinct loading paths). Their investigation encompassed CED, and SED, along with three established metal fatigue models—the Smith–Watson–Topper (SWT) parameter, the Chen–Xu–Huang (CXH) criterion, and the Fatemi–Socie approach. They concluded that the CED model identified plane-specific energy and gave better predictions than the SED model, while the SWT model, the CXH model, and the modified Fatemi–Socie model all gave effective predictions, especially the modified Fatemi–Socie model with the highest correlation coefficient.

A theoretically optimal damage parameter should possess reasonable physical meaning (theoretical validity), robustness (consistent performance across various conditions), and practical applicability (implementation feasibility) [13]. The application of CED presents specific computational challenges, requiring numerical integration across multiple material planes and specialized analytical tools. While simplified implementations exist for small-strain and linear elastic conditions, comprehensive analysis typically necessitates advanced computational platforms like Endurica [43,45] for accurate evaluation and implementation.

Seeking to enhance the theoretical basis of CED within a rigorous mechanical framework, Verron and Andriyana [13] developed a novel predictor based on Eshelby’s stress tensor theory for multiaxial fatigue analysis in elastomers. Their theoretical derivation established a fundamental relationship between energy dissipation mechanisms during micro-defect propagation and the characteristic properties of the configurational stress tensor. Specifically, the researchers formulated this predictor through the mathematical analysis of the minimal negative eigenvalue associated with the damage component of the Eshelby stress tensor, establishing an innovative configurational and mechanical approach for predicting fatigue-induced crack initiation in rubber materials. le Cam et al. [46] adopted minimum configurational stress [13] for damage assessment in carbon black-filled NR under both uniaxial and complex multiaxial loading scenarios. Andriyana et al. [47] compared the predictor based on configurational mechanics approach (CMA-predictor) to the critical plane approach (CPA-predictor) of Saintier et al. [48]. They found that both the predictors gave good results based on the experimental data [17], where the CMA-predictor demonstrated superior predictive accuracy, especially in torsion predominant loading conditions. Their results show that the CMA-predictor could be a promising way to treat the fatigue problems of rubber. However, the computational implementation of the CMA-predictor, specifically regarding its cyclic accumulation and incremental evolution throughout fatigue loading cycles, remains an active area of research. In contrast, the CPA-predictor has achieved successful integration within finite element analysis platforms. Therefore, continued development in the numerical implementation of the CMA predictor and various experimental data are required in order to evaluate its efficiency.

Previati and Kaliske [49] utilized the CMA-predictor and other four different predictors including the maximum principal stretch, SED, maximum principal Cauchy stress, and material force based on fracture mechanics (very closely related to the so-called *J*-integral) to evaluate multiaxial fatigue loading in pneumatic tires. They reported that the five predictors mentioned previously are capable of identifying the same crack location in the tire section, which aligns with the results of a durability test conducted on the tire. Additionally, the CMA-predictor is the only one able to differentiate between different load cycles with the same maximum load, making it particularly useful when specific load cycles need to be examined in detail. However, its computation requires the relatively fine time discretization of the cycle, as the calculation involves considering damage accumulation throughout the cycle. On the other hand, the procedure for the computation of material forces is quite standardized where all the required input quantities, such as the deformation gradient, SED, Cauchy stress tensor, and the shape functions of the element, are known and quite simple to handle. Given that the material force closely resembles the *J*-integral, it is important to give further attention to this in future research studies.

Recently, Gosar et al. [50], building on continuous damage mechanics, proposed a novel multiaxial energy-based approach that incorporates mean stress corrections. This method combines elastic strain variations with complementary energy transformations to formulate a novel energy-based damage indicator. This innovative parameter demonstrated significant predictive accuracy in estimating the fatigue life characteristics of commercial vehicle air spring systems, with experimental validation showing strong agreement with measured data. They concluded that their energy-based damage parameter requires less computational effort compared with the CPA-predictor and also offers online fatigue damage calculation, meaning that damage is obtained continuously regardless of the complexity and length of loading histories. The damage parameter might be a potential candidate parameter for rubber fatigue but still needs to be evaluated under various types of loading conditions.

#### 2.1.3. Stress-Based Damage Parameters

The application of stress as fatigue damage in elastomeric materials has historically faced significant challenges, particularly during the early stages of rubber fatigue research. This limitation primarily stemmed from the prevalent use of displacement-controlled testing methodologies, which complicated precise stress estimation due to the inherent nonlinear constitutive behavior characteristic of rubber materials under finite deformation. However, recent studies suggest that stress-based damage parameters may be better suited to characterize the fatigue properties of rubber [51,52,53]. Their experimental observations revealed that under the comprehensive consideration of large deformation conditions, crack propagation patterns consistently aligned with the orientation of peak first principal stress achieved during one loading cycle, even under complex non-proportional loading scenarios [53].

For rubber materials, each principal strain corresponds to a principal stress. As for stress-based damage parameters, the maximum principal Cauchy stress, which is the maximum eigenvalue of the Cauchy stress tensor, usually indicated by $\sigma_{\max}$, is commonly utilized in rubber fatigue literature. André et al. [51] established a fundamental relationship between crack propagation orientation and stress distribution patterns, demonstrating that crack planes consistently are perpendicular to the maximum principal Cauchy stress direction under simple torsion. Their findings indicated that this stress-based parameter could serve as an effective local descriptor for quantifying multiaxial fatigue damage mechanisms in NR materials. Saintier et al. [53] identified maximum first principal Cauchy stress as a superior damage quantification parameter, demonstrating its capability to precisely predict crack orientation across the diverse fatigue loading scenarios studied. Furthermore, this stress-based approach offers significant advantages over energy- or strain-derived criteria, particularly in its ability to provide realistic fatigue life predictions under hydrostatic stress conditions, thereby eliminating the theoretical limitation of infinite fatigue life predictions associated with alternative methodologies—something that contradicts experimental data. Verron and Andriyana [13] assessed the effectiveness of the maximum principal Cauchy stress using uniaxial and equiaxial experimental data from four different types of rubber materials—filled and unfilled NR compounds, filled and unfilled SBR compounds—provided by Roberts and Benzies [21]. It was found that the maximum principal Cauchy stress gave better overall predictions compared to the maximum stretch ratio and SED, particularly for both filled and unfilled NR. Tao et al. [54] used stress mean and stress amplitude as inputs of various analytical constant life diagram (CLD) models to predict the fatigue life of carbon cord-reinforced hydrogenated nitrile butadiene rubber (HNBR) composites under non-relaxing uniaxial tensional loading conditions. Their comparative analysis revealed that both the enhanced Harris CLD model [55] and piecewise linear CLD [56] demonstrated satisfactory predictive accuracy when validated against experimental fatigue life data.

Nevertheless, Abraham et al. [6] concluded that the maximum principal engineering stress (alternatively known as Cauchy or nominal stress) failed to serve as a reliable indicator for estimating the fatigue life of filled EPDM and filled SBR compounds. This is because, for these non-strain crystallizing rubbers, the fatigue life increases as the minimum stress is raised, while maintaining a constant stress amplitude. Poisson et al. [36] conducted a comparative analysis of fatigue behavior in polychloroprene rubber (CR) specimens under both uniaxial and biaxial loading conditions, revealing that the first principal stress criterion exhibited limited effectiveness in fatigue life prediction accuracy.

Although the orientation of crack planes generally aligns perpendicular to the principal stress direction, the maximum stress alone cannot account for the increased life when the minimum stress in the cycle rises at the same level of maximum stress. It also fails to differentiate between uniaxial and biaxial loading. Additionally, for certain load paths, the maximum stress may remain constant even as the loading conditions change [11].

The maximum Cauchy stress behaves differently in fatigue prediction across various rubber-like materials and loading conditions, prompting researchers to continue evaluating its effectiveness in different types of rubber materials. Saintier et al. [48] employed the first and second invariants of the Cauchy stress tensor as damage parameters. However, they revealed that the prediction accuracy of fatigue life was limited for NR materials subjected to multiaxial loading. Instead, it was found that CPA method, considering both damaged-end reinforcement mechanisms, was able to predict non-proportional multiaxial fatigue life, as well as to locate the crack initiation site and orientation, including internal cracks. The main drawback of this approach is its inability to take into account the size/gradient effects.

Luo et al. [57] developed a three-dimensional (3D) effect stress idea by taking into account the three principal Cauchy stress ranges, which was subsequently implemented in various industrial vibration-damping elastomeric components [58,59,60,61]. Shangguan et al. [62] applied multiple damage parameters for an industrial elastomeric component and revealed that stress-based parameters exhibited superior accuracy compared to both strain-based and energy-based parameters.

Since strain or energy density parameters fail to account for the influence of hydrostatic stress on lifetime, Brunac et al. [63] introduced a new multiaxial damage predictor based on the Dang Van sphere and the Haigh diagram. This method involves considering the closed path defined by the varying stress tensor in the 6D stress space and determining the center and radius of the Dang Van sphere to define the mean stress and stress amplitude of an ‘equivalent’ 1D cyclic loading. This new approach has significant improvements over previous methods, including the CED [35] and the effective stress [48], and overcomes the limitation of strain-based and energy-based predictors. Further experimental comparisons are however needed to evaluate the model, particularly for cases where the conventional Haigh diagram is not applicable.

Based on continuous damage mechanics (CDMs), Ayoub et al. [64] proposed an equivalent stress parameter (a CDM-predictor) based on the extension of the CDM model in [65] and then proposed another three-dimensional model coupled with the CED [35] to assess the fatigue life of SBR under combined tension and torsion loading with constant and variable amplitudes [12,66]. The developed CDM/CED model, which incorporates a fatigue damage parameter coupled with an accumulative damage rule, is suggested to provide a satisfactory agreement with experimental data under complex fatigue loading conditions. Its effectiveness was further examined by considering the effect of load ratio and was verified by Ayoub et al. [66].

The concept of effective or equivalent stress is based on the assumption that the material’s stiffness deteriorates as it degrades. Similarly, using the “loss of load-bearing capacity” of rubber as an indicator of failure is a common approach in many studies [27,37,67,68,69]. However, Rangarajan and Ramarathnam [70,71] experimentally found that the variation in stiffness of filled NR was not monotonic with fatigue life and the stiffness loss demonstrates limited reliability as a damage parameter. They revealed a phenomenon of stiffness restoration during specific phases of the fatigue process which cannot be captured by CDM theory. While the fatigue life prediction using the well-known CDM approach is satisfactory, the model’s prediction of stiffness degradation differs significantly from experimental observations. This indicates that it is challenging to correlate residual life or life consumed with the fatigue-induced reduction or changes in stiffness. These finding prompts researchers to reconsider the approach to rubber fatigue damage.

#### 2.1.4. Discussions on Potential Abilities of Various Damage Parameters

The evolution of fatigue damage parameters from strain-based to energy-based and then to stress-based reflects the rising complexity of mechanical fatigue loading conditions experienced by rubber compounds. The mechanical fatigue loadings of interest range from uniaxial to multiaxial conditions and from constant amplitude to variable amplitude, as well as comprising varying load mean values or load ratio under uniaxial loadings. Fatigue under these loading conditions may induce unique damage mechanisms. For instance, the major difference related to damage mechanisms between uniaxial to multiaxial conditions is that rotations of principal stress/strain or material plane orientations during cyclic multiaxial loading conditions exist, which highly influence the damage accumulation rules in the prediction models.

In many engineering materials that behave elastically and linearly (such as metals under small strains), principal stress and principal strain directions are aligned. However, rubber materials often exhibit complex behaviors that can cause misalignment due to rubber’s nonlinear, anisotropic, and viscoelastic properties, particularly under large deformations or complex loading conditions such as combined axial and shear loads [72].

Although numerous damage parameters have been developed to assess dynamic fatigue in rubber materials, it is widely understood that no single universal failure criterion applies to all types of rubber or engineering materials under various loading conditions. Typically, a robust multiaxial fatigue model must exhibit load phase sensitivity, mean stress dependence, variable amplitude adaptability, and capable of accounting for crack closure effects [72]. Some researchers have employed mixed damage parameters to model rubber fatigue. For instance, Harbour et al. [16] employed normal strain components for critical plane identification while utilizing the CED on the determined plane for fatigue life prediction, thereby enhancing the CED’s effectiveness.

In general, several key factors—such as strain crystallization, crack closure under compression, hydrostatic compression due to the (nearly) incompressible nature of rubber materials, rotations of principal stress or strain, and rotations of the material plane due to finite strains—significantly influence the fatigue life of rubber-like materials in various ways. These factors should be incorporated into the selected damage parameters when using the crack nucleation method. For instance, regarding strain-crystallizing rubber like filled NR, fatigue life is enhanced with elevated minimum strain levels (characterized by increased *R*-ratio values). Furthermore, compressive loading in a primary loading direction frequently coincides with complex multiaxial stress conditions, involving concurrent shear and tensile components in orthogonal orientations. The maximum principal strain and SED may not be accurate for compressive loading predictions as crack closure was not included.

Moreover, limited fatigue data and significant variability within the data itself further complicate the accuracy of rubber fatigue life prediction, especially to multiaxial fatigue life. Although a number of damage parameters are used to represent multiaxial fatigue data, none seem robustly successful in achieving consistent correlation, especially in unifying multiaxial data, which necessities more verifications and proposals in a wider range of rubber compounds, loadings, and temperatures as well as environmental conditions.

#### 2.1.5. Applications for Crack Nucleation Method

One of the ultimate goals of rubber fatigue research is to improve fatigue resistance and accurately predict the fatigue life of rubber components. Rubber components, primarily made from natural or synthetic rubber, are widely used in various fields, including aviation, automotive, and rail transportation, due to their energy-absorbing capabilities and elastic reversibility. Tires and engine/transmission mounts are typical examples of rubber components in the automotive industry. These components play a crucial role in absorbing energy, vibration, and sound caused by the excessive movement of the vehicle during operation. In service, rubber components are often exposed to complex multiaxial loadings, making them susceptible to fatigue failures. Therefore, fatigue failure is a critical concern in elastomer design.

**Figure 3:**
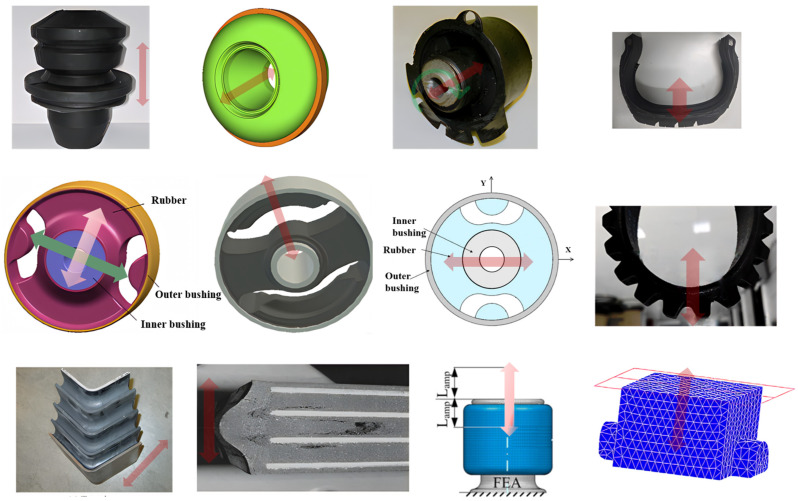
Typical rubber components available in the literature for fatigue life prediction, where the arrows indicate directions of the global loads applied to these components: (**a**) an automotive jounce bumper [68]. Reproduced with permission from Elsevier publisher. Copyright 2011; (**b**) a metacone component [61]. Reproduced with permission. Copyright 2006, Elsevier Ltd.; (**c**) a passenger vehicle cradle mount [72]. Reproduced with permission. Copyright 2013, Elsevier Ltd.; (**d**) cross-section of 175/75R14 tire [75]. Licensed under a Creative Commons Attribution License (CC BY 4.0); (**e**) a rubber mount [67]. Reproduced with permission. Copyright 2009, Elsevier Ltd.; (**f**) a roll front component [27]. Reproduced with permission. Copyright 2008, Elsevier Ltd.; (**g**) a rubber component [76]. Reproduced with permission. Copyright 2016, John Wiley and Sons Ltd. (**h**) R1 type toothed V-belt [77]. Reproduced with permission. Copyright 2018, Elsevier Ltd.; (**i**) a Chevron rubber spring [60]. Reproduced with permission. Copyright 2009, Elsevier Ltd.; (**g**) laminated elastomeric bearing pad [41]. Reproduced with permission. Copyright 2021, Elsevier Ltd.; (**k**) air-spring [78]. Reproduced with permission. Copyright 2013, Elsevier Ltd.; (**l**) an engine rubber mount [26]. Reproduced with permission. Copyright 2004, Elsevier Ltd.

Typical rubber components used in the literature for fatigue life estimation based on the crack nucleation method are summarized in Figure 3, along with the directions of the global loads applied to these components. Various researchers have employed diverse damage parameters for fatigue life assessments in rubber components, achieving satisfactory results from an engineering perspective.

Although the maximum principal strain criterion does not predict the reported differences between simple and equi-biaxial tension, it remains in use today, particularly for rubber components in engineering. Samad et al. [68] applied the maximum principal strain to predict fatigue life of an automotive jounce bumper (including 90% of NR and 10% butadiene rubber), and the comparison between the predicted and experimental results, including fatigue life and the crack nucleation area, showed good agreement. Li et al. [67] also used the maximum principal strain to evaluate the fatigue life of rubber engine mounts and discovered that the predicted fatigue lives agreed well with the test results. Similarly, Woo et al. [27,73] and Kim et al. [26] employed the maximum Green–Lagrange strain to calculate the fatigue lives of several different elastomeric components, such as a roll front component [27], a transmission mount [73], and an engine rubber mount [26]. The predicted and experimental fatigue lives of the rubber component correlated within a two- to four-fold accuracy range. It should be noted that Kim et al. [26] found via experimentation that the fatigue lives of filled NR specimens and mounts decreased at the same load amplitude as the tensile mean load increased by. This suggests that positive mean stresses are more detrimental to the fatigue life of NR compared to zero or negative mean loads. This finding contrasts with the results from other studies [14,19,53,74].

Many engineers and researchers have also used SED for correlating computational predictions with experimental fatigue life data [8]. Kim et al. [26] also used the maximum SED to predict the crack nucleation life and found SED is less correlation with the fatigue life than the maximum principal Green–Lagrange strain. Li and Xin [77] utilized maximum SED as a fatigue damage parameter, successfully correlating toothed V-belt bending fatigue predictions with experimental data from De Mattia tests within a two-fold accuracy range. Wang et al. [75] used the maximum SED range as the damage parameter to determine the fatigue life of tire bead and proved that experimental results of the 175/75R14 tire were consistent with the estimated value, which showed a good correlation.

Rubber components frequently experience complex multiaxial stress states under compression, where primary compressive loads induce secondary tensile and shear stresses in orthogonal directions, except under pure hydrostatic conditions. This stress complexity necessitates careful consideration in fatigue analysis, as crack initiation predominantly occurs on planes experiencing shear/tension rather than pure compression. Consequently, fatigue criteria neglecting crack closure effects, such as maximum principal strain or SED, often prove inadequate for compression-dominated loading scenarios [79].

Elastomeric components often experience complex multiaxial loading with variable amplitudes, where peak stress and strain components typically occur asynchronously due to phase differences in loading cycles. Zarrin-Ghalami and Fatemi [72] employed a computationally efficient CPA using maximum normal strain planes and CED-based damage assessment to predict fatigue life and crack orientation in vehicle cradle mounts under various axial torsion loading scenarios (in-phase and out-of-phase, constant and variable amplitude loading). Their approach demonstrated that the method could achieve similar results with the critical CED criterion while significantly reducing computational load by eliminating exhaustive plane-by-plane calculations. Several works [72,80,81] employed the CED theory to predict the nucleation life of automotive rubber components (e.g., tire, busing, and mount).

Luo et al. [60,61] utilized a 3D effective stress (a function of three principal stress ranges) to evaluate the crack initiation of several rubber components, including a metacone type of rubber spring and a rubber-to-metal-bonded Chevron rubber spring, which are anti-vibration components used in rail vehicle suspensions. The component fatigue tests and simulation results demonstrated strong correlation using this methodology.

Recently, some researchers have focused on critical plane approaches (CPA), where a critical plane serves as the plane where a crack is expected to nucleate and grow. The critical plane can be identified by maximizing a fatigue damage parameter based on energy, stress, or strain measures. Oman et al. [78,82] employed a CPA method proposed by Fatemi and Socie [83] to estimate the fatigue damage of an air-spring utilized in truck-cab and proved its success in consistence with the experimental results. But the authors point out that the estimated damage is very conservative due to the large scattering of the experimental results, and that more experimental data are needed to increase the rate of confidence and other parameters for damage calculation will have to be examined. Chung and Kim [76] applied the CHX model [84] and a modified rain-flow counting algorithm for multi-components into the fatigue life prediction of a rubber mount under random loadings and found that the numerical results matched with experimental data with uniaxial and multiaxial loadings.

While various damage parameters, including the maximum principal strain, SED, equivalent stress, and CED, are employed for rubber fatigue life prediction, their accuracy remains geometry- and loading-specific. Challenges persist in validating these methods under general service conditions, particularly due to limited multiaxial data. Additionally, crack nucleation approach would require long fatigue testing time to obtain the damage parameter versus fatigue life data across geometries, often yielding significant data scatter.

### 2.2. Crack Growth Method

The fatigue crack initiation methodology is commonly employed to predict the fatigue life required for microscopic defects to develop into macroscopic cracks with a critical length of approximately one millimeter. It is often applied in situations where micro-cracks dominate the overall fatigue life. On the other hand, the crack growth approach focuses on fatigue life estimation from macroscopic crack propagation to final failure, particularly applicable when macro-crack growth dominates the component’s total life [72]. Furthermore, in the fatigue crack propagation approach, the prior imposition of a crack in the sample eliminates the random crack initiation and improves reproducibility [85], which is of great importance, especially in terms of the large scattering of rubber fatigue data.

From the crack propagation point of view, the driving force to initiate a crack growth is the energy required per unit area of the newly formed crack surface, which is called the strain energy release rate (ERR) [86], *G*, or “tearing energy” (TE) in elastomers specifically [32], *T*; it is expressed as [87]:

(8) $$T=-\frac{\partial U}{\partial A}$$

In this formulation, parameter *A* represents the cross-sectional area of an individual crack surface, while *U* quantifies the total strain energy accumulated within the structural component.

To estimate the fatigue life of a rubber component using the fatigue crack propagation approach, the prior determination of the relationship between crack growth rate (d*a*/d*N*), where *a* denotes crack length and *N* represents the number of cycles, and the maximum tearing energy *T*_max_ is required. In this methodology, the maximum tearing energy *T*_max_ severs as the driving force of crack growth in rubber-like materials.

**Figure 4:**
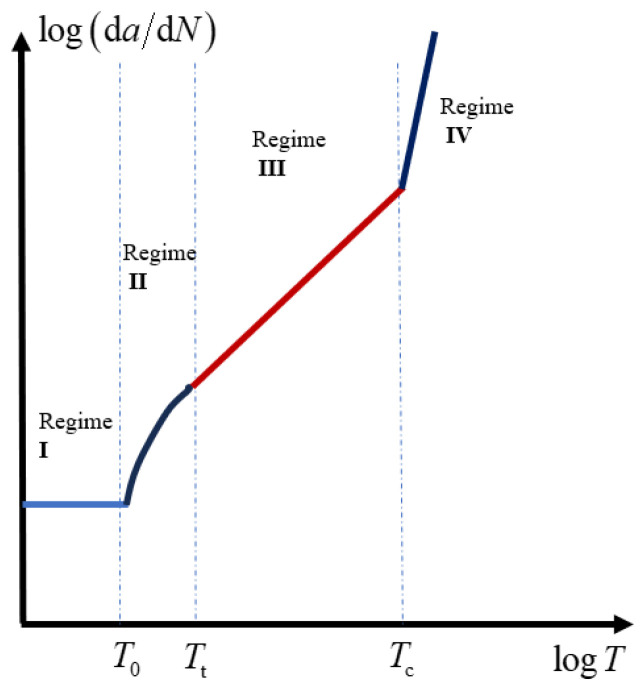
Four regimes of fatigue crack propagation characteristics in rubbery materials.

As for the general case of rubber rupture, Rivlin and Thomas [32] established that the correlation between crack growth rate and strain energy release rate is an intrinsic material characteristic, demonstrating invariance across different loading configurations and geometric parameters of test specimens. Fatigue crack growth behavior can typically be categorized into four distinct regimes (shown in Figure 4), based on the maximum tearing energy per cycle [88,89,90]. Regime III, which follows a power–law relationship, is commonly used to fit experimental crack growth data, allowing for the extraction of the constants *B* and *β*, such that:

(9) $$\frac{da}{dN}=BT^{\beta }$$

Having obtained the coefficients *B* and *β* for a rubber “material”, fatigue life in a rubber “component” can be computed using the integration of Equation (9) and the dependency of tearing energy (*T*) on the crack length (*a*):

(10) $$N=\int dN=\int_{a_{0}}^{a}\frac{da}{BT^{\beta }}$$

In cases involving geometrically complex components under multiaxial loading conditions, this dependency can be routinely captured through two distinct methodologies: an approximate fracture mechanics approach for specific loading scenarios [91] or advanced finite element-based crack propagation simulations that incorporate fracture mechanics principles, such as *J*-integral analysis [87], for the precise modeling of crack growth in arbitrary geometries under defined loading conditions.

For some specific specimens, the tearing energy can be calculated through short-cut formulas (shown in Section 3.2.2), which are frequently employed to attain the fatigue crack properties of rubber materials in laboratory. It should be noted that the available expressions for *T* would have limitations, e.g., the formula for the single-edged notched tension (SENT) specimen is for moderately large deformation (below nominal strains of 100 percent) and small cracks of length a, i.e., a/*w* << 1. For a much wider range of crack lengths and strains, the work carried out by Liu et al. [92] is suggested.

A number of studies [20,85,93] have discussed theories of fracture mechanics and crack growth in both metals and rubbers. For a more thorough understanding, one can refer to additional works [8,9]. The following section focuses on two main topics: the calculation of tearing energy and methods for determining the initial crack position and growth direction. Application examples from typical rubber components are also explored.

#### 2.2.1. Calculation of Energy Release Rate or Tearing Energy

For rubber specimens with complex geometries and loadings (e.g., industrial rubber components), it is challenging or impossible to find analytical solutions for tearing energy *T* equivalent to those available for simple specimens described in Section 3.2.2.

Thanks to the development of large deformation FEA, the technique for predicting fatigue failure using crack growth approach has been dramatically extended. As of now, the strain energy release rate can be computationally determined for arbitrary component geometries containing cracks under specific loading configurations [87]. Finite element analysis provides several alternative computational approaches for determining the strain energy release rate, encompassing the energy difference method [94] (also known as local/global energy balance [95], node release, or virtual crack extension technique [87]), *J*-integral formulation, and crack tip closure analysis. Busfield et al. [96] confirmed the numerical consistency of these methods, even when analyzing complex three-dimensional crack configurations. The choice of implementation strategy is therefore primarily determined by computational practicality and specific application requirements.

Regarding a global energy balance approach, the difference in the total strain energy of a structure with slightly varying crack lengths is calculated to estimate the ERR or TE. Some researchers [87,97] adopted the energy balance technique to estimate the TE of rubber components, including a gearbox mount and a cylindrically shaped suspension component, respectively. The researchers determined the strain energy release rate (ERR) or tearing energy (TE) by computing the difference in the internal strain energy (d*U*) between two fixed-displacement models with incrementally different crack surface areas (d*A*), expressed as d*U*/d*A*. This methodology demonstrated predictive accuracy within a two-fold range across various displacement conditions and deformation modes.

The technique used by Busfield et al. [87,97], similar to the “global” complex finite element method described by Ytuarte et al. [95], replicates all the degrees of freedom in a finite element model to store information related to TE. As a result, this analysis introduces a considerable computational overhead. Alternatively, Ytuarte et al. [95] proposed a local-based complex-variable finite element method, where complex-valued calculations are only conducted at a small group of elements surrounding the crack tip, so as to improve the computational efficiency of the fracture analysis.

The *J*-integral method [98] is other feasible way to estimate the TE in nonlinear problems. In the literature on rubber, the ERR and the *J*-integral represent the same quantity [99]. The *J*-integral defined by Rice [100] characterizes crack growth in nonlinear elastic rubbers. The *J*-integral characterizes crack tip stress–strain field intensity, analogous to the stress intensity factor *K* in linear elastic fracture mechanics (LEFMs). This path-independent parameter maintains consistent values when evaluated along arbitrary contours surrounding the crack tip in homogeneous materials. The crack is assumed to grow when the *J*-integral reaches a critical value, *J*_c_, which is measured experimentally. The *J*-integral has been proven to be equivalent to TE [101].

Today, the *J*-integral is available in most commercial finite element software and has been extensively used to analyze fractures of rubber or elastic and other stretchable materials [102,103,104,105]. For the evaluation of *J*-integral, ABAQUS adopts the energy domain integral method [99]. Tavosi et al. [106] calculated the *J*-integral using the finite element modeling of rubber stator in the commercial code ABAQUS/Standard and captured the dependency of TE on the crack length, where the initial crack with a length of 0.5 mm was gradually raised to a length of 1.5 mm and the TE was accordingly calculated. The initial crack position and direction were determined through repeated searching for probable orientation with the highest values of *J*-integral. However, the component’s life results have not been experimentally validated; instead, they only provide a relative life comparison between different types of rubber liners.

Peter et al. [107] calculated the ERR of NR using a well-established material force method, very similar to the well-known *J*-integral method and different only in the integration algorithm, within the Ansys 2020 R2 software. Due to limitations in the microscopic observation during the tested ozone, only surface crack length is measurable with sufficient accuracy, while its depth remains unknown. To address this, it is assumed that the cracks possess a semi-elliptical shape. Additionally, the cracks were assumed to be straight for simplification purposes.

One of the main advantages concerning the choice of the *J*-integral method is the fact that the *J*-integral can be evaluated independently of the stress field singularity near the crack tip and that it can be numerically computed without any great difficulty [108]. Unfortunately, the *J*-integral cannot predict the crack propagation direction [109].

For relatively complex components, researchers utilize approximate formulations for the strain energy release rate. For example, Lindley and Stevenson [91] applied simplified fracture mechanics to estimate the fatigue behavior of compression-loaded engineering mounts. Despite the approximation, the fatigue life estimates were found to be of appropriate magnitude accuracy. Recently, Mars and Fatemi [110] investigated three approximate formulas (11a–c) to estimate TE in terms of three different continuum mechanical parameters, including SED, CED, and the maximum principal strain, in combination with the classic relation for the ERR of a single-edge notched tension (SENT) specimen (described in detail in Section 3.2.2). They suggested the estimations are limited to crack size smaller than 6 mm and no of crack interactions with specimen boundaries.

(11a) $$T_{w}=2kWa$$

(11b) $$T_{W_{c}}=2kW_{c}a$$

(11c) $$T_{\varepsilon }=C\varepsilon^{2}a$$

where the factor *k* is determined empirically and given in Section 3.2.2; ε is the maximum principal strain; *C* represents a material-specific constant, sharing dimensional units with SED and exhibiting dependence on Young’s modulus (*C* = 9 MPa in their work); and W and $W_{c}$ represent SED and CED, respectively.

Similarly, Ding et al. [111] applied the equivalent stress proposed by Luo and Wu [61] to characterize the multiaxial stress state of rubber components and then substituted it as an alternative input form of the classic TE relationship for SENT in terms of stress measurement.

Approximate formulas are useful in engineering applications, but some researchers continue to explore novel alternative solutions for tearing energy, such as those proposed by Aït Hocine and Naït Abdelaziz [109].

#### 2.2.2. Analyzing the Susceptible Position and Direction for Crack Growth

While some researchers argue that the crack growth approach is suitable for cases where rubber components must withstand crack propagation before failure [112], many studies [94] have adopted it due to its feasibility, reproducibility, and efficiency. In any fatigue analysis using the crack growth approach, the most important decision is to identify the location where the cracks are most likely to initiate and the direction in which they are likely to grow [94]. The crack propagation approach applies when initial crack geometry, position, or specific dimensions under cyclic loading are well-defined.

For the initial crack information, a small pre-crack or some cracking experience is often applied. For instance, a controlled pre-crack was introduced at the outer surface center of the gearbox mount using razor blade incision during fatigue experiments [97]. On the other hand, the rubber–metal bond interface is identified as the primary fatigue crack initiation site, supported by experimental observations and preliminary FEA of the crack-free model for a suspension component [87].

However, in real rubber parts, it is often difficult to predict the exact location of crack initiation and crack propagation direction when using fatigue crack growth approach, and this step is crucial for calculating the required TE. To address this, some researchers approach the issue by considering several potential positions and directions during the finite element modeling process. Mirza et al. [94] suggested cracks are most likely to occur at locations where highest maximum principal strains occur. They established the FEA model of a spherical rubber bearing and carried out common stress analysis as the first step to narrow the range of possible crack nucleation and confirm its ozone using observed fatigue failure locations of the studied rubber component in experiment or in service. Based on this information, cracks of idealized geometry were modeled at four different locations (crack 1—a circumferential surface crack; crack 2—an inward surface crack; crack 3—an internal crack near interface; and crack 4—an internal crack at interface) around the confirmed ozone, and then the locations and direction of the cracks were identified by analyzing the relationship between the estimated TE and the crack size for different crack models. They concluded that crack 3—an internal crack near the rubber/inner interface—was the most likely to initiate and propagate along the circumference and would also extend toward the surface.

**Figure 5:**
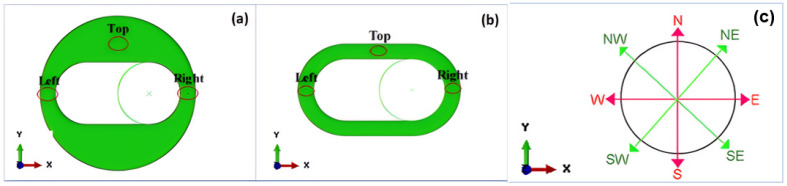
Susceptible positions selected for the analysis of the crack growth: (**a**) a conventional rubber liner, (**b**) a uniform wall thickness rubber liner; and (**c**) hypothetical directions for choosing the most susceptible crack growth direction [106]. Reproduced with permission. Copyright 2024, Elsevier Ltd.

Tavosi et al. [106] employed two steps to determine the crack position and growth orientation. Firstly, they divided the model geometry of the rubber stator (a 2D plane-strain domain due to symmetry) into three probable regions, “left”, “top”, and “right”, as depicted in Figure 5a,b, and the location with the highest possible *J*-integral was reasonably assumed to be the most susceptible position for the crack growth. Secondly, one end of the crack was fixed at the location with the highest *J*-integral, as identified in the analysis of the first step, and the other end was positioned at various directions, with each separated by 45 degrees from the previous (shown in Figure 5c). The direction with the highest *J*-integral values was then considered the most probable growth direction. To establish a relationship between TE and crack growth, the initial crack with a length of 0.5 mm was gradually raised to a length of 1.5 mm, and the crack driving force or TE was accordingly calculated.

When searching for the appropriate crack information, multiple simulations are required, leading to a considerable computational load. Based on the experimentally determined crack lengths, Peter et al. [107] assumed that the cracks possess a semi-elliptical shape and are straight on the specimen surface during the calculation of the ERR of NR for simplification purposes. Additionally, two models—one complete specimen without any cracks and a sub-model with a reduced length of 3 mm, instead of the original 100 mm, including the entire group of cracks—were combined to optimize the total computation time. To further minimize the computational cost, finite element mesh simplification techniques such as considering symmetry [87,97], plane strain [106], or stress conditions were used. The critical loading scenarios are also employed to identify the region where cracks are most likely to grow and to predict the direction of crack growth [94].

#### 2.2.3. Crack Growth Approach Applications

**Figure 6:**
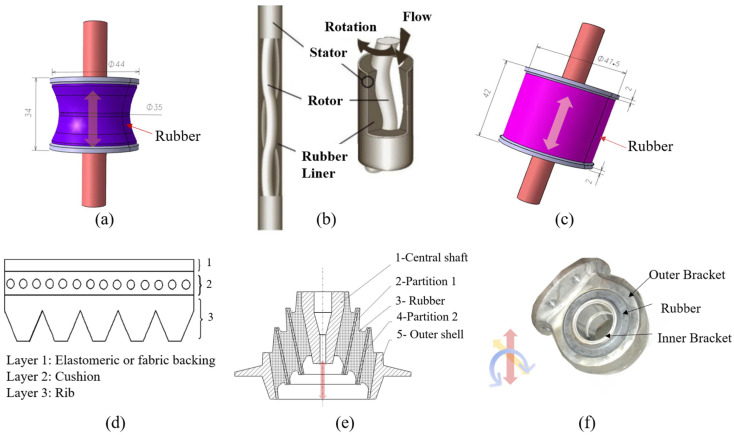
Typical rubber components available in the literature for fatigue life prediction using crack growth approach, where the arrows indicate directions of the global loads applied to these components: (**a**) a 3D elastomeric components. Redrawn by the authors based on the original data from Ref. [97]; (**b**) sketch of rubber stator, rotor, and rubber liner of 1/2 downhole motors [106]. Reproduced with permission. Copyright 2024, Elsevier Ltd.; (**c**) a cylindrical rubber component used in suspension system. Redrawn by the authors based on the original data from Ref. [87]; (**d**) typical rubber v-ribbed belt composition [99]. Reproduced with permission from Elsevier publisher, copyright 2007. (**e**) a cone-shaped rubber spring. Redrawn by the authors based on the original data from Ref. [111]. (**f**) a spherical rubber bearing. Modified by the authors using data originally from Ref. [94].

Several typical application examples using crack growth approach can be found in the literature [87,94,106,113], with the corresponding rubber components and global loadings presented in Figure 6. For instance, Busfield et al. [87,97] adopted the approach to estimate the number of cycles needed for crack growth between specific lengths in a gearbox rubber mount and a cylindrically shaped suspension component. The predicted data showed strong agreement with experimental results, demonstrating higher accuracy across a broader range of crack lengths, from the initial stage to the point of failure. Mirza et al. [94] estimated the fatigue life of a rubber spherical bearing utilized in railway suspension applications. They were not only able to successfully implement a fracture mechanics approach to fatigue but they also succeeded in redesigning the part and improving its durability and fatigue life. Also, Sundararaman et al. [99,114] investigated the fatigue life for v-ribbed rubber belts by simulating the dependency of energy release rate on the crack length in several areas prone to crack initiation. In the past decade, application using crack growth method in rubber components has been relatively limited [106,113]. For instance, Tavosi et al. [106] calculated the fatigue life of rubber stator of downhole motors by combining the crack growth rate and tearing energy (TE), derived from the *J*-integral method, using the finite element analysis of the ABAQUS/Standard.

One major difficulty in using the crack growth method for rubber parts is the requirement for prior knowledge of the initial crack’s location and condition, which ultimately leads to failure. However, such information is often unavailable and does not precisely reflect what the designer aims to evaluate. The evolving geometry of the crack introduces additional complexity. The numerical applications of direct fracture mechanics methods are both labor-intensive and computationally expensive, highlighting the urgent need for efficient and adaptable algorithms to simplify crack growth analysis in rubber components.

### 2.3. Fatigue Phase-Field Method

To obtain more accurate fatigue damage parameters, it is essential to develop a deeper understanding of fatigue mechanisms. The crack growth method based on classical fracture mechanics has been found to exhibit mesh dependency during numerical simulations, require additional crack propagation criteria, and become ineffective for modeling complex multi-crack interactions [115]. This has led to the exploration of new approaches for understanding the fatigue mechanisms of rubber-like materials.

Recently, an important method from fracture mechanics, considered by some research studies as a subset of continuum damage mechanics, known as the phase field method, was developed and employed to simulate the fracture, nucleation, and growth of fatigue cracks in rubber-like materials [116,117,118]. Such an approach eliminates the need to predefine crack locations and avoids additional complexities in modeling crack nucleation, branching, and coalescence [117], simplifying the analysis of both crack initiation and growth without extra technical requirements. Based on this model, the crack path is automatically determined by the principle of total potential energy minimization, without the need for arbitrary assumptions [119].

To the best of the authors’ knowledge, researchers [116,117,118,119] primarily utilize phase-field models to capture mechanical response evolution during cyclic loading and to analyze rupture or fracture properties of rubber-like materials, which provides a novel theoretical path to understanding fatigue mechanisms. Miehe et al. [120] were the first to investigate the phase field modeling of fractures in rubber-like polymers at finite deformations. Since then, many researchers [117,118,121,122] have extended the phase field method to estimate mechanical response during cyclic loading up to final failure. Among them, Loew et al. [117] established a fatigue phase-field model from mechanical energy balance to describe rubber damage under cyclic loading at finite strains. They introduced a fatigue damage source linked to accumulated viscous dissipation to achieve this. They concluded that the model is able to predict load cycle failure with a good agreement with the experimental data of a single-edge notched tensile (SENT) experiment and with the fatigue crack growth law of a pure shear specimen. They suggested that such a model still needs more experimental validation for complex specimens under multiaxial loading.

However, the fatigue phase-field approach is relatively complex, requiring an evaluation of its potential performance across a broader range of loading conditions, as well as demanding substantial computational resources, which hinder their applications in rubber components.

### 2.4. Aspect of Probability and Statistics Analysis

It is well recognized that data obtained from rubber fatigue tests exhibit a relatively large amount of scattering even in well-controlled experiment conditions [123], which is partly attributed to variations such as in material properties [124], microstructures, or the geometric properties of a specimen, modeling errors, and measurement conditions. The fatigue life models mentioned above are all deterministic, often providing a model or diagram based on 50% reliability by default. Consequently, their use in practical applications requires additional considerations to establish suitable safety margins for design (e.g., a factor of 2). Alternatively, probabilistic fatigue life models can incorporate various sources of uncertainty [125,126,127], enabling reliability analysis to enhance deterministic predictions—particularly when addressing practical engineering challenges—on a more rational foundation.

Belkhiria et al. [126] developed a probabilistic method to estimate the fatigue reliability of rubber components under pure tension or pure torsion cyclic loading. This approach combines Wohler diagrams, in which SED is used as the damage parameter, with the ‘strength–load’ method coupled with Monte Carlo simulation (MCS) technique. This probabilistic model incorporates variations in Wohler material parameters, rubber material types, and applied loads by assuming normal distributions for these factors. Thereafter, in the year of 2023, the same group extended the work to multiaxial probabilistic fatigue reliability of rubber parts using CED as the damage parameter [127]. Such a probabilistic methodology requires limited computational effort, making it a practical tool for engineers to solve problems and to more effectively predict the fatigue limit in numerous rubber-related engineering applications with greater confidence.

The idea that the fatigue life of metal-like materials obeys lognormal distribution is widely recognized in the engineering community. However, there are few studies on the probabilistic distribution function of rubber fatigue life based on the relevant fatigue test data [128], since many differences in fatigue behavior, such as strain crystallization, exist in rubber-like materials [7,53,63,129]. Dizon et al. [130] claimed that the Weibull distribution function adequately describe the fatigue data of rubber compounds obtained from artificially notched dumbbell specimens, but they did not give evidence of the goodness of fit. Very recently, Wang et al. [128] calculated the goodness-of-fit of the Kolmogorov–Smirnov (K–S) statistic results using three distribution models, including normal, lognormal, and two-parameter Weibull distributions, for three kinds of NR test data (a total of 20 datasets). They concluded that the Weibull distribution is the best choice to describe the rubber fatigue life data. Based on the Weibull distribution, probabilistic fatigue life (*P*–*ε*–N or *P*–*S*–*N*) curves of the studied rubbers were obtained through two approaches, including sample information aggregation principle for small samples and a power–Weibull statistical model for complete data.

Larin [123] developed a probabilistic model for fatigue damage accumulation, which is based on CDM and takes into account the random variation in fatigue curve parameters. They suggested that fatigue life should be best approximated by either a lognormal or Weibull distribution, which qualitatively aligns with experimental data from flat rubber specimens under cyclic tensile loading. However, the rubber material compound is not provided in the work.

Very recently, Liu et al. [131] investigated the probabilistic fatigue life of vulcanized NR, using uniaxial tension tests on dumbbell-shaped specimens and coupling three distribution models (normal, lognormal, two-parameter Weibull distributions) with a support vector machine model to extend sample data. They found that solely the lognormal model is proved most effective for mixed datasets of measured and extended data. Based on this conclusion, the same group [132] subsequently developed a probabilistic uniaxial fatigue life model (*P*–*S*–*N* curve), which accounts for the strain ratio effect using an equivalent strain amplitude and employs the group method to determine the *P*–*S*–*N* curve.

Yaagoubi et al. [133] proposed a probabilistic life prediction approach based on the Monte Carlo simulation (MCS) procedure and crack growth approach. In this approach, initial crack length follows a Weibull distribution representing the size of agglomerates, while the failure crack length corresponds to the diameter of the test specimens (dumbbell and concave specimens described in Section 3.2.1). The predicted curves of the three studied load levels for both specimen types exhibit excellent agreement with the measured values.

It can be inferred from the published works that diverse probabilistic predictive models can be constructed by combining uncertainties from various fatigue-influencing factors with the deterministic models discussed earlier.

### 2.5. Data-Driven Method Based on Machine Learning

Although remarkable achievements have been obtained in previous research studies, difficulties in constructing a clear multifactor mathematical model using the conventional fatigue life estimation models still exist and the prediction accuracy of the models needs to be further improved. With the advancement and popularization of machine learning (ML) algorithms, artificial intelligence methods have recently been extensively used to evaluate the fatigue life of metal-like materials [134,135], and their application to predicting the fatigue performance of rubber-like materials has been found in several research studies [105,136,137,138,139,140,141].

An integrated approach combining an enhanced sparrow search algorithm with support vector regression (ISSA-SVR) was used to predict rubber uniaxial fatigue life under various strain ratios [140]. The comparison of predicted and experimental fatigue data shows that the ISSA-SVR model delivers the highest accuracy, with results falling within 1.5 times the scatter band. Meanwhile, an extreme learning machine-based model was developed to predict the fatigue life of NR specimens, incorporating factors such as peak engineering strain, temperature, and material hardness [139]. It turns out that the extreme learning machine model outperforms both the backpropagation neural network (BPNN) and SVR models in terms of efficiency and accuracy, achieving correlation coefficients of 0.9898 and 0.9802 for training and test samples, respectively, when comparing predicted and actual fatigue lives. Using the same experimental data by Duan et al. [142], another BPNN model with an improved sine–cosine algorithm (ISCA) for optimizing the model’s parameters was established [138]. It was found that the BPNN model obtained by the ISCA was more accurate in predicting rubber fatigue life, in which both training and testing samples fall within 1.5 times dispersion line.

It appears that there is an optimal ML algorithm to evaluate the fatigue life of rubber materials under uniaxial loading, taking various factors into account [105,136,141]. This highlights the potential for obtaining extended test data [131] using ML approaches, especially when working with small sample data, which is common given that gathering comprehensive fatigue data for rubber materials is time-consuming, costly, and often impractical. For example, Liu et al. [131] utilized a SVR model to extend sample data and subsequently evaluated three distribution models for NR materials. It should be noted that they all focus on NR materials. Marco et al. [37] performed multiaxial fatigue experiments on tungsten nanoparticle-reinforced polychloroprene rubber (CR), and then employed six machine learning models for fatigue life assessments. Their findings revealed that the deep neural network achieved the highest accuracy, with an average prediction error of 14.3%.

It is believed that an increasing number of studies will focus on predicting rubber fatigue life based on various ML algorithms with the development and diversification of learning algorithms. Indeed, while the ML-based approach is capable of establishing the relationship between fatigue life and certain fatigue-influencing factors, and especially suitable to expand the data especially under small samples, it still depends, however, on certain appropriate damage parameters and cannot reveal the fatigue damage mechanics of rubber.

## 3. Fatigue Experiments of Rubber-like Materials

Fatigue research is an experiment-based science, and the validation and analysis of fatigue life modeling for rubber materials necessitates corresponding fatigue experiments. Due to their hyper-elastic and viscoelastic nature, rubber materials exhibit strong sensitivity to variations in loading modes, stress and strain amplitudes, frequencies, strain rates, waveforms, and temperatures [6]. Parameters affecting the constitutive response can directly or indirectly influence the fatigue resistances.

According to the review by Mars and Fatemi [3], the primary factor influencing fatigue properties is the mechanical loading history imposed on rubber materials or components. This section thus addresses recent advances in the fatigue experiments of rubber-like materials and components, considering various characteristics related to the mechanical loading. It should be mentioned that to avoid misunderstanding and clarify easily, in the following discussions, the general term “load” or “loading” will be utilized to represent a type of mechanical severity under fatigue or cyclic loading as quantified by various parameters, including strain-based, stress-based, and energy-based parameters, to name a few.

In general, uniaxial and multiaxial fatigue loads are two major classes of loads that exist in the literature on rubber fatigue. Uniaxial or simple loads are usually characterized by load limit parameters such as maximum, minimum, mean, *R*-ratio, and amplitude (alternating or range), which are interdependent, meaning that any two of them uniquely determine the remaining parameters. These parameters for characterizing uniaxial loads were illustrated clearly in the review by Mars and Fatemi [3], however, there are no definitions for multiaxial loads, which is relatively complex to define in detail.

From aspects of stress or strain state of a material point at a specific instant, there are only three states, composed of uniaxial, biaxial, and triaxial stress or strain states. Furthermore, in the uniaxial case, the direction of the principal stress and strain remains constant [143]. However, for multiaxial cases, the direction of principal stress or strain may shift relative to the material’s orientation during cyclic loads, which will lead to various fatigue damage accumulations. Additionally, it is widely known in rubber fatigue studies that shear and tension loads result in different modes of cracks (e.g., mode I, II, III, or mixed modes), while slight compressive loads have no impact on crack initiation or growth. In fact, under larger compression conditions, the cracks seem to initiate in the middle of the cylindrical hourglass-shaped specimens and propagate in the loading direction [144]. Various combinations of shear and tension loads have also been designed in rubber specimens, such as proportional or non-proportional loads. The crack orientation also depends on the loading conditions, e.g., a crack propagates perpendicular to the loading direction in tension fatigue tests.

### 3.1. Considering Various Mechanical Loading Histories

In the rubber literature, various forms of mechanical loads are frequently employed in fatigue experiments, consisting of uniaxial tension/compression (also called simple tension/compression), simple shear (also called torsion, twist), and biaxial tension as a typical type of multiaxial loads. Clearly, further research is required in this area for more comprehensive multiaxial fatigue experiments. Biaxial tension loads can be categorized into equi-biaxial, inequi-biaxial, in-phase, out-of-phase, proportional, non-proportional loads, or a combination of these. These characteristics are summarized in Table 1. It is important to note that other aspects of mechanical load history, such as prior load history (e.g., the sequence of high and low severity events, prolonged periods of rest), loading rate or frequencies [54], and waveform shape, are essential. For a deeper understanding, readers can refer to the research studies by Mars and Fatemi [3] and Tao et al. [54].

Mechanical loading history plays a critical role in fatigue analysis of all engineering materials. Key parameters such as frequencies, stress and strain ranges, strain energy, and energy release rate (ERR) must be considered when defining loading conditions. While loading frequency has slight impact on the fatigue behavior of strain-crystallizing rubbers, it significantly affects amorphous rubbers due to time-dependent crack growth [89]. At high frequencies and strains, rubbers are susceptible to thermal runaway, where rapid temperature rise leads to material degradation [145]. The influence of waveform on fatigue properties is more pronounced in amorphous rubbers compared to strain-crystallizing elastomers, though this varies based on polymer and filler composition [7].

Biaxial loads and triaxial loads (involving three independent axes of stress/strain) are the most commonly discussed types of multiaxial loads and are frequently encountered in practical applications, although multiaxial loads can theoretically involve more than three axes. In particular, 3D stress states can represent complex loading scenarios in real-world situations, such as components subjected to combined loads (bending, torsion, axial loads). However, 2D states (plane stress or plane strain) are more commonly used in rubber fatigue analysis to achieve highly efficient computations. To date, fatigue test results primarily focus on uniaxial tension, simple shear, or combined tension–torsion loads.

#### 3.1.1. Uniaxial Loads

In uniaxial tension, a material is subjected to a load along a single axis, causing it to stretch in that direction while the other dimensions may contract. This type of load is commonly analyzed in structural and material engineering to understand how materials behave under tension. In uniaxial tension, the stresses or strains are applied along a fixed axis. Conducting fatigue tests on rubber specimens under constant amplitude and uniaxial load conditions is relatively straightforward, as there are established international standards, such as ASTM D4482 [146] and ISO 6943 [147], to guide the process.

**Figure 7:**
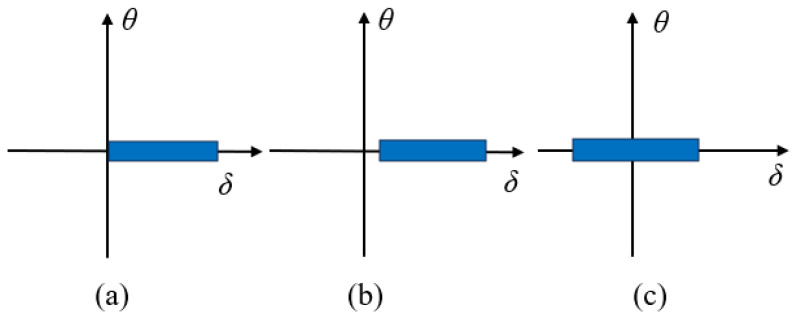
Uniaxial loading paths (*δ* = displacement; *θ* = twist angle): (**a**) fully relaxing loads; (**b**) non-relaxing loads (*R_δ_* > 0); (**c**) compression loads (*R_δ_* < 0).

Fully relaxing loads” is like a technical term, which means a specified type of loading, the fatigue life and the maximum strain or strain amplitude exhibit a strong power–law relationship for rubber-like materials [18]. Under such loads, the fatigue life increases as the strain amplitude decreases, which is well known in rubber fatigue fields [17]. However, rubber components frequently experience a significant static load combined with smaller dynamic loads. In these cases, the load is rarely fully relaxed, indicating that the minimum load or *R*-ratio is typically non-zero. In fact, the minimum or mean loads significantly influence the fatigue resistances of rubber materials, especially for certain strain-crystallizing rubber materials [3]. Furthermore, tracking the state of embedded cracks through the instantaneous material configuration, open or closed, is very important to determine whether strain-crystallization will occur. To investigate the strain crystallizing, uniaxial loads, characterized by constant amplitude or maximum loads while varying the minimum load, mean load, or *R*-ratio, are designed to monitor the behavior of rubber specimens. To study the crack closure effect, compressive loads are frequently applied. Such experiments are often carried out on smooth rubber specimens in a laboratory. Uniaxial loads with an *R*-ratio of 0 are referred to as fully relaxing loads or fully unloading conditions, while non-relaxing loads correspond to negative or positive *R*-ratios. Three typical uniaxial loads are illustrated in Figure 7.

**Figure 8:**
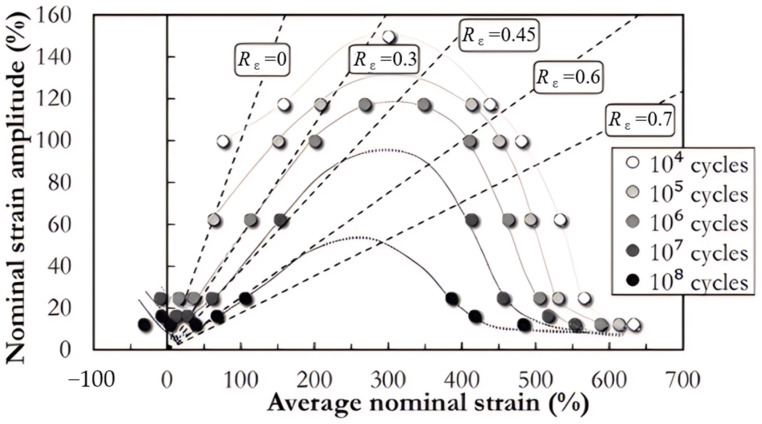
Haigh diagram based on the original results of Cadwell et al. [14] for fatigue data of filled NR under non-relaxing uniaxial tension [148]. Reproduced with permission from Elsevier Ltd., 2021.

**Figure 9:**
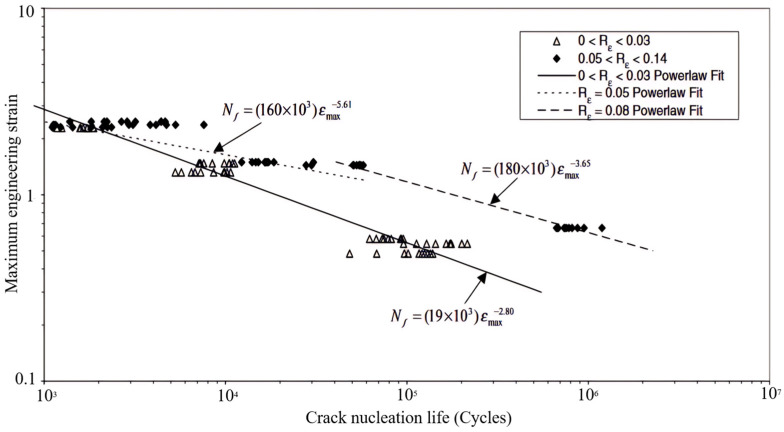
Effect of positive *R* ratio on fatigue nucleation life [129]. Reproduced with permission from John Wiley and Sons, 2003.

**Figure 10:**
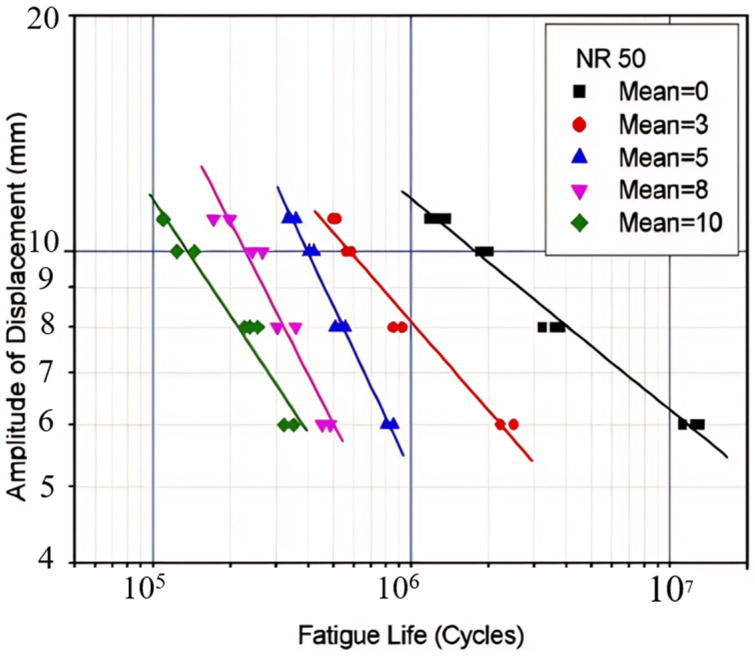
Displacement–fatigue life curve [27]. Reproduced with permission from Elsevier Ltd., 2008.

**Figure 11:**
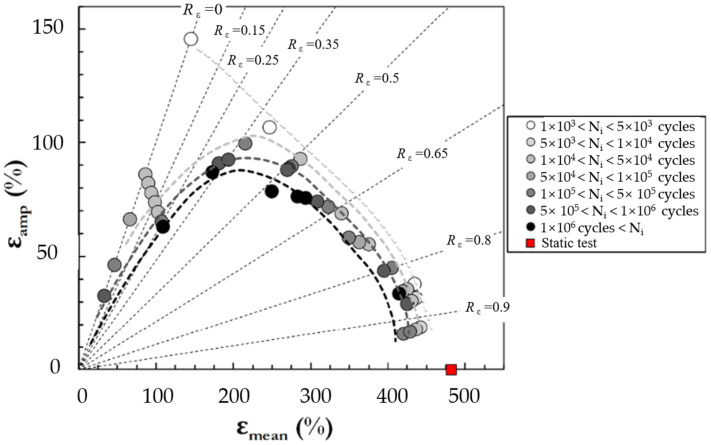
Haigh diagram plotted in the mean maximum principal nominal strain versus maximum principal nominal strain amplitude space [145]. Reproduced with permission from Elsevier Ltd., 2021.

To investigate the strain crystallizing effect on rubber fatigue behavior, several researchers [14,31,34,36,66,129,148,149,150] developed fatigue experiments in terms of crack initiation approach and crack growth approach using various rubber compounds. Regarding aspects of crack nucleation approach, Cadwell et al. [14] and Fielding [19] analyzed fatigue performances with several types of rubber materials using cylindrical dumbbell and ring-shaped test specimens. The most important data from the experiments are redrawn in a Haigh diagram by Champy et al. [148], as shown in Figure 8. They found that the fatigue life of NR and Butyl-B material increases when the minimum strain increases up to the maximum value, even though the strain range is kept constant. Beatty [151] expanded on the work of Cadwell et al. [14] by studying SBR, a non-strain-crystallizing material, and observed that the fatigue life of SBR slightly declines as the positive minimum load increases during the loading cycle. Mars and Fatemi [129] performed experiments on both fatigue macro-crack nucleation and crack growth in filled NR material using simple tension and planar tension specimens. Their results showed that a small positive *R* ratio can greatly improve fatigue life and reduce crack growth rates, especially at lower strain ranges (see Figure 9). However, Woo et al. [27] observed that the fatigue life of a filled NR material decreased as the mean displacement increased, while the displacement amplitude remained constant, as shown in Figure 10. This result appears to contradict the reinforcement associated with strain-induced crystallization mentioned in previous studies. This is likely due to the fact that the results only covered a small portion of the full Haigh diagram. To demonstrate the fatigue performances of crystallizable rubber under a large positive load ratio range, Champy et al. [148] created a full Haigh diagram (for tension–tension loads), as shown in Figure 11, with well-controlled experimental conditions, and they observed that strain-induced crystallization provides reinforcement at lower load ratios (up to a displacement load ratio of 0.35). However, at higher load ratios, this reinforcement effect diminishes, resulting in a bell-shaped Haigh diagram, consistent with the findings of Cadwell et al. [14].

**Figure 12:**
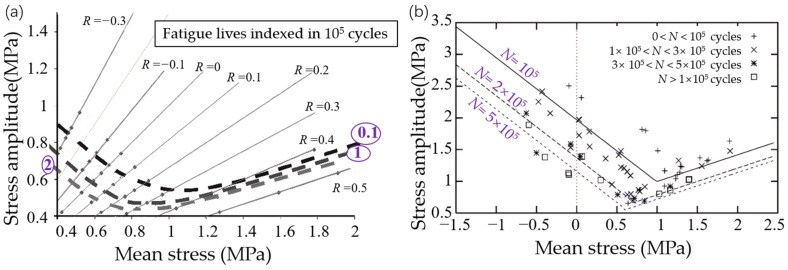
Fatigue test results under non-relaxing uniaxial tensional loads in terms of Haigh diagram: (**a**) polychloroprene rubber [36]. Reproduced with permission from Elsevier Ltd., 2011; (**b**) filled NR [150]. Reproduced with permission from Elsevier Ltd., 2011.

Poisson et al. [36] created a tension Haigh diagram of the polychloroprene rubber (CR) based on experimental data (shown in Figure 12a), where the mean of the axial component of the first Piola–Kirshoff stress tensor and the amplitude section are displayed on the abscissa and ordinate, respectively. They found that the tension fatigue behavior of CR can be divided into two distinct regions, separated by the *R* = 0.2 curve. In the first region, fatigue life decreases with an increasing *R*-ratio, consistent with the characteristics of amorphous rubber; in the second region, the trend is reversed. Moreover, under an *R*-ratio of 0.5, fatigue life exceeds that under *R*-ratio of 0.4 by more than 10 times. These variations in fatigue life might arise from the potential crystallization of CR materials. Similar test data for other rubber compound can also been found in Ayoub et al. [66], who carried out 52 tests on AE2 specimens (depicted in Section 3.2.1) for filled SBR materials under load paths similar to those shown in Figure 7b.

**Figure 13:**
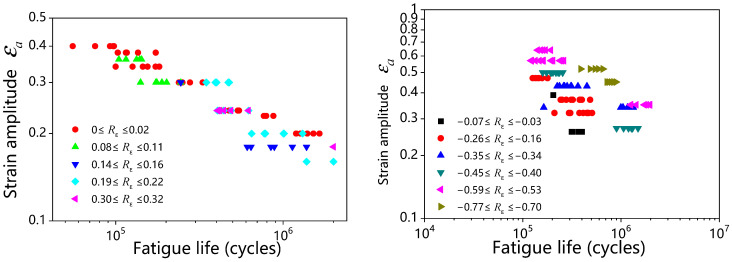
Fatigue life dataset of filled NR under negative and positive strain *R*-ratios [31]. Reproduced with permission from John Wiley and Sons, 2014.

The Haigh diagrams mentioned above are all based on tension–tension loads, where negative loads are not considered. To address this, Oshima et al. [74] conducted fatigue tests on vulcanized NR used in automotive engine mountings under various negative and positive stress *R*-ratios and created a Haigh diagram. Their findings also revealed that positive mean stress induces strain crystallization, which results in an extended fatigue life for vulcanized NR. Similar results were obtained by Saintier et al. [150] to reveal the positive stress ratio generated reinforcement mechanisms of strain-induced crystallization, as shown in Figure 12b. Wang et al. [31] published uniaxial fatigue life data against the strain amplitude under a wide range of strain *R*-ratios including negative and positive *R*-ratios for a type of filled NR (see Figure 13). Such experimental data were also applied by other researchers [29,105] to evaluate their approaches to predict rubber fatigue life.

**Figure 14:**
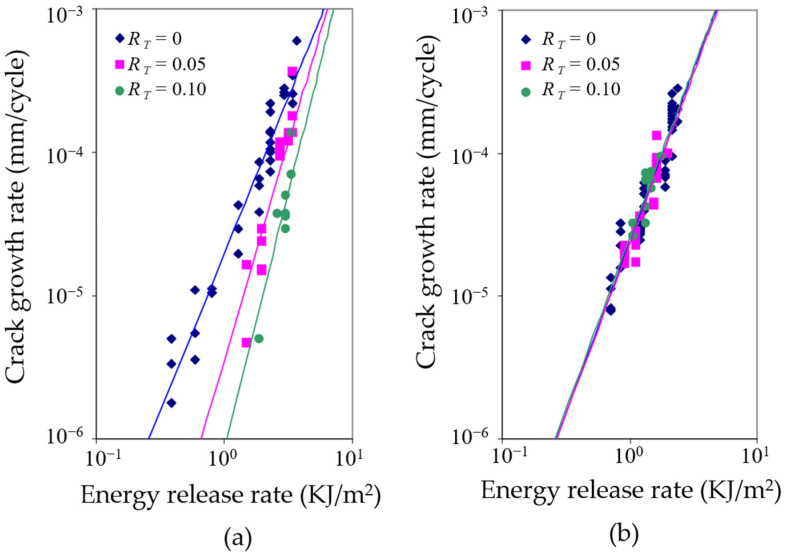
Crack growth rates of NR and SBR specimens under constant amplitude loading at varying *R*-ratios for (**a**) NR and (**b**) SBR [152]. Reproduced with permission from John Wiley and Sons, 2017.

On the other hand, several studies [129,152] have investigated strain crystallization impact on fatigue crack growth behavior under non-relaxing loads. Harbour et al. [152] used pre-crack pure shear specimens to assess the fatigue crack propagation properties of two common rubber compounds, NR and SBR. Tests were conducted at tearing energy *R*-ratios of 0, 0.05, and 0.10 for both materials. The crack growth rates under constant amplitude loading for NR and SBR specimens are presented in Figure 14, show that strain-crystallizing compounds like natural rubber are highly sensitive to changes in the *R*-ratio, while non-crystallizing compounds like SBR remain unaffected by it. These findings emphasize the importance of considering the material type when predicting fatigue behavior.

Based on the results above, a consistent conclusion can be drawn that moderate positive minimum loads are beneficial in enhancing the fatigue resistance of strain-crystallizing compounds like NR materials to some extent, but they are either harmful or have no significant effect on SBR materials. While many experimental data have focused on the strain crystallization effect, especially under tension–tension loads, a unified model to accurately quantify this effect on the fatigue life of rubbers is still under development [128,138,152,153]. It should be mentioned that Abraham et al. [6] found that in some non-strain crystallizing rubbers, fatigue life may increase with higher minimum stress at constant stress amplitude. Unlike NR, this effect was linked to filler systems rather than strain crystallization.

#### 3.1.2. Simple Shear Loads

Simple shear, also referred to as torsion or twist load, is a type of mechanical loading in which a material or structure undergoes deformation due to a force that causes sliding layers or particles relative to each other, without significantly altering the material’s volume. In the case of a cylindrical object, such as a shaft or rod, subjected to torsion (twist) load, one end is rotated around the longitudinal axis while the other end is either held fixed or is rotated in the opposite direction. This results in shear stresses along the material’s cross-section, often referred to as pure shear when the material experiences shear stress without any accompanying normal stress.

**Figure 15:**
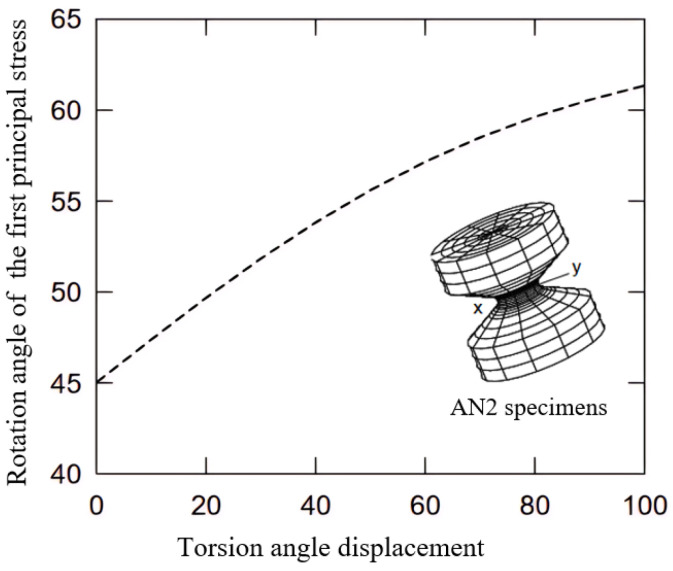
First principal stress direction rotation observed during pure torsion testing of AN2 specimens (unit: degree) [53]. Reproduced with permission from Elsevier Ltd., 2006.

It is important to mention that simple shear loading involves stress acting on a surface, typically in two perpendicular directions; this differs from the uniaxial loading, which is confined to a single direction along one axis. In other words, simple shear loading can be considered a form of biaxial loading, but it specifically refers to the conditions under which shear is applied. Because of finite strain behavior, pure torsion tests do not achieve a true pure shear strain condition [17]. Even a simple torsion test on filled NR, which involves large strains and incompressibility, may induce significant rotations of the principal stress directions (see Figure 15), meaning it should be treated as a non-proportional fatigue test [53].

**Figure 16:**
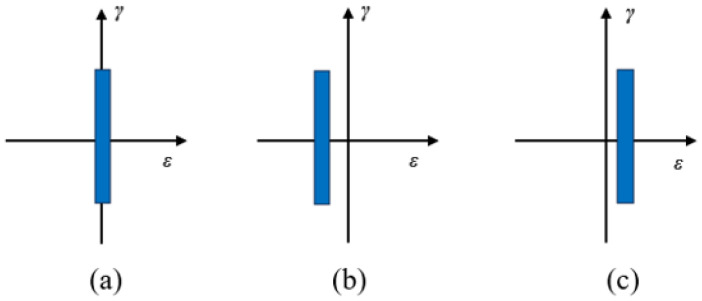
Simple shear loading paths with (**a**) no static axial preload, (**b**) compressive axial preload, (**c**) tensional axial preload, where *γ* = shear strain; *ε* = normal stain. Created by the authors based on the dynamic fatigue results on double-shear rectangle specimens [14].

With regard to fatigue tests under simple shear loads, several research groups [14,17,23,39,48,53,64,154] provided valuable test data for various rubber compounds. Cadwell et al. [14] measured the fatigue life of rubber under simple shear loads as well as combined axial and shear loads (the loading paths are depicted in Figure 16) at various levels of maximum and minimum strain using a double-shear rectangle specimen (depicted in Section 3.2.1). It covered nine loads, including three levels of shear strain ranges (−25% to 25%, 0% to 50%, and 75% to 125%) and three levels of static normal strain (−12.5%, 0%, 25%).

**Figure 17:**
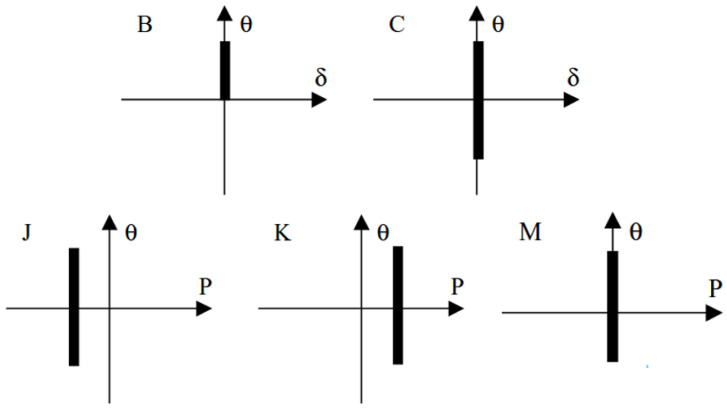
Pure torsion loading paths: *δ* = displacement; *θ* = twist; *P* = axial load; and *T* = torque. Extracted from the original figure in Mars and Fatemi [17]. Reproduced with permission from John Wiley and Sons, 2005.

Mars and Fatemi [17] carried out 91 fatigue tests in a wide range of strain histories including pure axial, pure torsion, proportional axial torsion, and non-proportional axial torsion for filled NR using axisymmetric specimens made of a rubber ring bonded between two steel mounting rings (depicted in Section 3.2.1). Five loading paths (as shown in Figure 17) were designed for pure torsion, and the loading details are summarized in Table 2. Similar load paths were also investigated by Harbour et al. [16]. It can be seen from their tests that the load *R*-ratios (*R_θ_*) are limited at 0 and −1, and more test data are supposed to add to the examination of the effect of *R*-ratios on fatigue life.

Saintier et al. [48,53] carried out fatigue tests considering tension–compression and pure torsion loads for vulcanized NR using three types of cylindrical hourglass-shaped specimens (named ‘Diabolo’, AN2, and AN5, indicating that the notch radius is equal to 1.75 and 4.75 mm, as depicted in Section 3.2.1). Their experimental data on fatigue crack initiation in rubber under various loading conditions has been used in various studies [39,47,63] to evaluate new multiaxial fatigue damage parameters.

Zine et al. [23,39] carried out uniaxial and biaxial fatigue experiments for a SBR compound using a uniaxial tension specimen and a pure shear specimen with a circular hole in the central section to enforce the crack nucleation localization. Ayoub et al. [64] carried out fatigue tests under uniaxial tension (17 levels; *δ*_max_ = 5.6~28 mm; *R_δ_* = 0) and pure torsion loads (like path B in Figure 17; 10 levels; *θ*_max_ = 100~145° with intervals of 5°; *R_θ_* = 0) on AE42 specimens (depicted in Section 3.2.1) for a SBR material under constant amplitude loading conditions. Subsequently, the same group [66] carried out another 13 pure torsion tests (*θ*_max_ = 40~100° with intervals of 5°; *R_θ_* = 0) on AE2 specimens (depicted in Section 3.2.1) for the same rubber compound. Using the same testing device, Hottin et al. [154] carried out multiaxial fatigue tests involving different cyclic loadings (tension, torsion, and combined tension–torsion) for the filled NR compound. The fully relaxing pure torsion tests include four levels (*θ*_max_ = 60~150° with intervals of 30°) for AE2 specimens and four levels (*θ*_max_ = 90~180° with intervals of 30°) for AE42 specimens, respectively.

Fatigue data under simple shear loads for filled NR and SBR materials exist, but they are mainly focused on the fully relaxing shear loads. To fulfill the gap for filled SBR materials, Ayoub et al. [66] further investigated *R* ratio effects on pure torsion fatigue tests and performed 33 tests (divided into six groups with *θ*_max_ = 55~145°; *θ*_min_ = 5~70° with the reciprocal of *R_θ_* ratio falling within 1.43~21) for non-relaxing pure torsion loadings on AE2 specimens. Further fatigue data under non-relaxing shear loads for various rubber compounds are needed in future research to better understand their behavior and improve fatigue life predictions.

#### 3.1.3. Multiaxial Loads

To extend the warranty period of rubber components and enhance the analytical models used for predicting their fatigue lives, an enhanced understanding of the role of loading conditions in fatigue behavior, particularly under multiaxial cyclic loads, is essential [16,38]. Regarding multiaxial loads (with biaxial tension being more commonly discussed in the literature), the specific load modes involve various aspects, including equi-biaxial and inequi-biaxial, in-phase and out-of-phase, and proportional and non-proportional fatigue loads, among others. Table 1 summarizes the characteristics of these multiaxial loads.

Under equi-biaxial loads, the material experiences equal stresses or strains along two different axes. The stresses or strains applied in both directions (e.g., X and Y axes) are identical in magnitude, although they may vary cyclically over time. The principal directions are aligned with the applied load directions and remain constant because the loading is symmetric. An equi-biaxial tension load can be taken as a type of proportional load. The differences between equi-biaxial and inequi-biaxial loads lie in the magnitudes of the two loads along two axes. The principal directions remain aligned with the loading axes, but due to the different magnitudes, the material experiences asymmetric stress states under inequi-biaxial loads.

Regarding in-phase loading, all stress or strain components vary in sync, meaning their peaks and valleys occur simultaneously, with no phase shifts between them—all reaching their maximum and minimum values at the same time. Under such loading conditions, the principal stress/strain directions remain constant throughout the loading cycle because the stress/strain components rise and fall together. The loading path is often a straight line or an ellipse. On the other hand, for out-of-phase loading, there is a time lag or phase difference between the stress or strain components. One component might reach its peak while another is at a different point in its cycle, which means that there is a phase shift between the stress or strain components and their peaks do not coincide. Under such loading conditions, the principal stress/strain directions rotate throughout the loading cycle so that the loading path is relatively complex.

Proportional and non-proportional loadings are also frequently found in the literature, especially for multiaxial loads. The stress or strain components under proportional loads maintain a constant relationship in terms of magnitude and direction throughout the loading cycle. The ratio between the different stresses/strains remains constant. The ratio of loads in different directions stays fixed, so the loading path is linear, and there is no change in principal stress/strain directions during the cycle even if the magnitudes vary over time.

Mars and Fatemi [17] proposed that proportional loading occurs when axial and torsional displacements maintain a linear relationship with a fixed gradient, initiating from the origin, whereas Saintier et al. [48,53] demonstrated that a simple torsion test induces strong principal stress direction rotation, which is this taken as a non-proportional fatigue loading. In non-proportional loading, the ratio between the stress or strain components changes over time. The relationship between stresses or strains in different directions varies during the loading cycle, leading to complex loading paths (e.g., spirals or irregular loops) and rotating principal directions. This might be the most damaging type of fatigue loading. Indeed, there are some overlaps between out-of-phase and non-proportional loads. For example, the presence of a phase shift, φ, between axial and torsional components typically characterizes non-proportional loading conditions, whereas the zero phase angle φ corresponds to proportional loadings [13,17].

To elaborate more clearly, a multiaxial load can be written as follows:

(12) $$\left\{\begin{array}{l} \lambda \left(t\right)=\lambda_{m}+\lambda_{a}\sin(\omega t)\\ \gamma \left(t\right)=\gamma_{m}+\gamma_{a}\sin(\omega t+\varphi ) \end{array}\right.$$

where λ, $\lambda_{m}$, and $\lambda_{a}$ represent the stretch ratio and the mean and its amplitude during one cycle, respectively; γ, $\gamma_{m}$, and $\gamma_{a}$ indicate shear strain (twist per unit length) and the mean and its amplitude during one cycle; ω is the signal frequency; and φ is phase angle between axial and twist.

If *R*-ratios for the axial and torsional directions are equal and φ=0, then

(13) $$\frac{\lambda \left(t\right)}{\gamma \left(t\right)}=\frac{\lambda_{a}\left[\frac{1+R}{1-R}+\sin(\omega t)\right]}{\gamma_{a}\left[\frac{1+R}{1-R}+\sin(\omega t)\right]}=\frac{\lambda_{a}}{\gamma_{a}}=\mathrm{const}.$$

which indicates that in-phase loads correspond to proportional loads.

If the *R*-ratios for the axial and torsional directions are equal and φ≠0, then

(14) $$\frac{\lambda \left(t\right)}{\gamma \left(t\right)}=\frac{\lambda_{a}\left[1+\sin(\omega t)\right]}{\gamma_{a}\left[1+\sin(\omega t+\phi )\right]}=f(t)$$

which reveals that out-of-phase loads correspond to non-proportional loads.

It should be noted that proportional non-relaxing loads will occur if the *R*-ratios for the axial and torsional directions are equal but not equal to zero. If the *R*-ratios for the axial and torsional directions are not consistent, in-phase loads might correspond to non-proportional loads.

In summary, equi-biaxial and inequi-biaxial loads differ in the magnitude of loads applied in two directions: equi-biaxial is balanced, whereas inequi-biaxial is not. In-phase and out-of-phase loads differ in timing (synchronization): in-phase is synchronized, whereas out-of-phase is asynchronous. Proportional and non-proportional loads differ in the relationship between load components: proportional has constant ratios, while non-proportional has variable ratios, often causing principal stress directions to rotate. Moreover, the terms “uniaxial load” or “multiaxial load” often refer to macroscopic or global forces or displacements applied in one or multiple directions. However, from a local perspective, the stress state at any material point is limited to a maximum of three-dimensional stress, regardless of the number of macroscopic load directions or displacements.

Generally, proportional and in-phase loading typically correspond to simpler multiaxial loads, wherein the principal strain directions remain unchanged throughout one cycle. In contrast, non-proportional and out-of-phase loading represent more complex multiaxial loads, during which the principal stress or strain directions rotate continuously throughout the cycle. In fact, due to factors such as the material’s constitutive behavior, the degree of deformation, and its geometry, even if a uniaxial global load is applied, the state of stress can be complicated, and the principal direction can be changed along the load path [48,53,76]. Similarly, for tensile–torsion tests, even large prescribed loading angles can produce almost uniaxial tensile tests along the gauge length [155].

**Figure 18:**
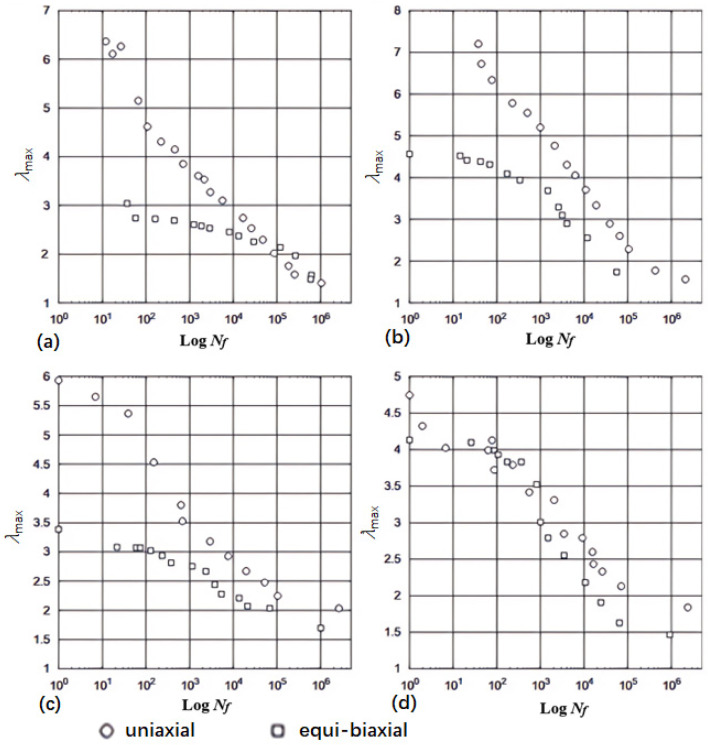
Comparative analysis of fatigue life data under uniaxial versus equi-biaxial tensile loading conditions in Roberts and Benzies [21], based on maximum stretch ratio (*λ*_max_) parameters: (**a**) filled NR, (**b**) unfilled NR, (**c**) filled SBR, and (**d**) unfilled SBR [13]. Reproduced with permission from Elsevier Ltd., 2011.

Comparisons between uniaxial and equi-biaxial extension fatigue end-of-life are very important since the two deformation modes for elastomers result in significant differences, not only for the stress–strain response but for the fatigue life. The most comprehensive equi-biaxial fatigue test for rubbers is from Roberts and Benzies [21], which can be found in the bibliography, and is utilized by some studies [13,20] as validation data to evaluate multiaxial damager parameters unifying uniaxial and equi-biaxial fatigue lives. Employing a multi-specimen membrane inflation system where four planar circular elastomeric membranes were simultaneously subjected to biaxial deformation, the equi-biaxial fatigue life calculated from the mean value of eight tests for a given stretch level was obtained [21]. The redrawn fatigue life data versus the strains of the four types of rubber compounds by Verron and Andriyana [13] are depicted in Figure 18.

**Figure 19:**
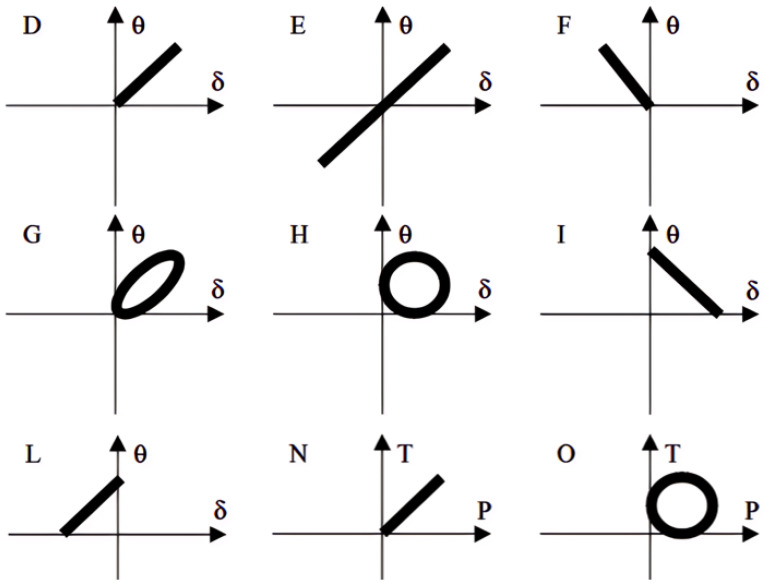
Axial torsion loading paths: *δ* = displacement; *θ* = twist; *P* = axial load; and *T* = torque [17]. Reproduced with permission from John Wiley and Sons, 2005.

Mars and Fatemi [17] performed comprehensive fatigue experiments in a wide range of strain histories, encompassing uniaxial tensile deformation, pure shear stress, proportional combined tension–torsion and non-proportional tension–torsion loading scenarios for filled NR. There are 15 distinct fatigue loading configurations, systematically designated from path A to O, which were characterized by varying *R* ratios, initial preloading conditions, and phase angles between loading components. The pure torsion loading paths (paths B, C, J, K, and M) are examined in Figure 17; therefore, only combined axial torsion loading paths are shown in Figure 19, and elaborations for each multiaxial paths are provided in Table 3. They took the inelastic response of rubber and the crack closure effect into consideration by investigating different loads. For instance, a comparison between force-controlled loading and displacement-controlled loading shows the effects of the permanent set and stress softening or the Mullins effect on fatigue properties. However, it can be seen that load ratios (axial displacement ratio *R_δ_,* twist angle ratio *R_θ_*) were designed at specific values of 0, *∞*, and −1 for combined axial torsion loads and that reinforcement related to strain-induced crystallization in multiaxial loadings is omitted.

To provide more multiaxial strain states, such as combined tension–torsion fatigue data, for a SBR material, Ayoub et al. [12] carried out comprehensive tension–torsion fatigue tests using a cylindrical hourglass-shaped specimens (called AE42, with curvature radii equal to 42 mm, described in Section 3.2.1) in an Instron-8874 servo hydraulic testing device. They performed 13 tests for proportional tension–torsion fatigue data (like path D in Figure 19, *R_δ_* = *R_θ_* = 0). The maximum axial displacement *δ_max_* falls within 5.6~25.2 mm with multilevel intervals of 1.4, 2.8, and 5.6 mm, and the maximum torsion angle *θ_max_* was designed with two values of 50 and 100 degree for the 13 tests. Subsequently, Ayoub et al. [66] utilized AE2 specimens (curvature radii equal to 2 mm) to carry out 12 proportional tension–torsion fatigue tests (like path D in Figure 19; *R_δ_* = *R_θ_* = 0; *θ_max_* = 40~80° with intervals of 20°; and *δ_max_* = 2.25~5.63 mm with intervals of around 1.1 mm).

Using the same type of testing device, multiaxial fatigue tests involving different types of cyclic loads (tension, torsion, and combined tension–torsion) and three specimen geometries were carried out on a filled NR [154]. The investigated specimens include AE2 and AE42 samples, which were subject to tension, twist, or combined tension–torsion, and a modified flat dumbbell specimen (the thickness equals to 2 mm) for uniaxial tension tests. They carried out nine proportional tension–torsion fatigue tests (like path D in Figure 19), including five tests (*R_δ_* = *R_θ_* = 0; *θ_max_* = 30~150° with intervals of 30°; and *δ_max_* = 2.5 mm) on AE2 specimens, and four tests (*R_δ_* = *R_θ_* = 0; *θ_max_* = 60~150° with intervals of 30°; and *δ_max_* = 6 mm) on AE42 specimens.

**Figure 20:**
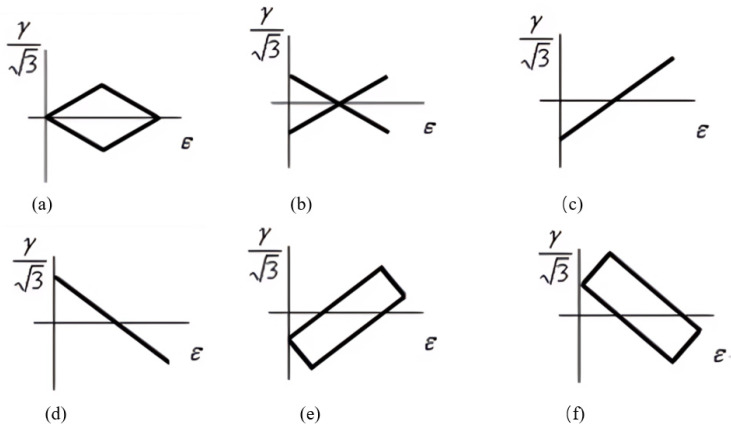
Proportional and non-proportional loading paths (combined tension–torsion) [156]. Reproduced with permission from John Wiley and Sons, 2008.

Wang et al. [156] designed several combined tension–torsion loading paths (as shown in Figure 20), which are significantly different from those in the literature [17,66]. They obtained a total of 10 multiaxial loads, with the shear strain range kept between −35% and +35% (*R**_γ_*** = 0) and the maximum normal strain falling within 100% nd 200% (*R**_ε_*** = 0), indicating limited data for each individual path.

**Figure 21:**
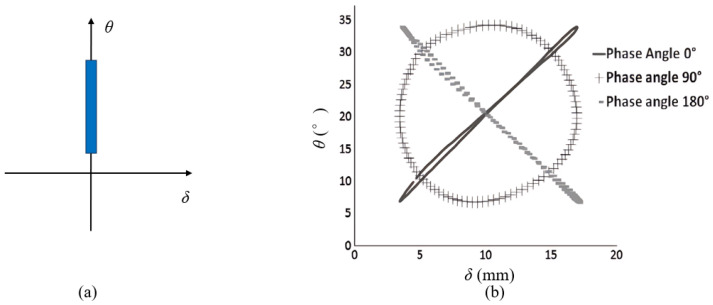
Fatigue loading paths: (**a**) non-relaxing pure torsion path [66]; (**b**) multiaxial fatigue paths (*δ* = displacement; *θ* = twist) [36]. Reproduced with permission from Elsevier Ltd., 2011.

It can be seen that load ratios (*R_δ_*, *R_θ_*, *R**_γ_***, *R**_ε_***) were designed at a specific value of 0 for combined axial–torsion loadings and that *R* ratio effects or reinforcements related to the strain-induced crystallization of rubber materials under multiaxial loads is not considered in such experiments. Although non-relaxing pure torsion loads (as shown in Figure 21a) were investigated in Ayoub et al. [66] for filled SBR compounds, the only fatigue data involving non-relaxing multiaxial (such as combined tension–torsion) fatigue tests can be seen in Poisson et al. [36], concerning a vulcanized polychloroprene rubber (CR) material. They conducted combined tension–torsion tests (illustrated in Figure 21b) with three phase angles (*φ* = 0°, 90°, and 180°) on dumbbell-type specimens (depicted in Section 3.2.1) using an electromechanical biaxial fatigue test device, BOSE. A more detailed analysis of fatigue behavior under multiaxial non-relaxing loads is needed in future research to better understand their impact on material performance.

Fatigue experiments should be simple, with well-defined and calculable or measurable stress or strain levels to obtain reliable results. However, multiaxial fatigue tests are currently relatively complex, and experimental loading conditions are currently chosen with no significant justification other than to cover “a wide range of strain histories” or “a wide range of fatigue lives” [157]. Consequently, it seems like there is no available general testing standard, even though most rubber components are subjected to multiaxial repeated loadings. To avoid some inherently invalid experiments, multiaxiality should be redefined from the local “material viewpoint” rather than the global machine perspective to quantize the strain and stress states induced by various loading conditions, which was recently highlighted in Ref. [157]. Additionally, multiaxial fatigue test data are relatively rare due to experimental complexity and extensive temporal investment necessary for acquiring the fatigue characterization datasets of elastomeric materials.

#### 3.1.4. Variable Amplitude Loads

The fatigue tests discussed above are based on constant amplitude loads, which are frequently utilized in fatigue studies; practical rubber components typically involve complex loading like varying amplitude conditions over time. Understanding the effects of variable amplitude loads is essential for enhancing the accuracy of fatigue behavior predictions in rubber components in realistic service conditions, where loading complexities significantly exceed the simplified constant-amplitude configurations typically implemented in experimental settings.

**Figure 22:**
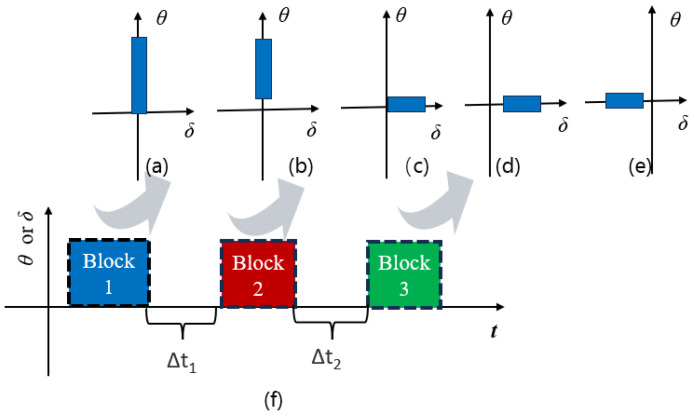
Evolution of variable amplitude fatigue loads (*δ* = displacement; *θ* = twist).

Characteristics of mechanical loadings that influence the fatigue performances of rubber discussed in the literature include load levels, load *R*-ratios, compression loads leading to crack closure effects, load sequences, and load directions, to name a few. Therefore, variable amplitude fatigue loads can have a large number of combinations, as shown in Figure 22. In this figure, each block (shown in Figure 22f) represents a type of constant amplitude fatigue load, of which uniaxial tension and pure torsion are typical simple constant amplitude modes (depicted in Figure 22a–e; they can also be combined into axial–twist loading paths). The dwell period (Δt_1_ or Δt_2_) is also an important factor in crack growth process especially for some rubber compounds like filled SBR material [152]. Different numbers of repeated times of constant amplitude loads in each block and the load levels of the amplitude itself, can cause complicated combinations of variable amplitude loads.

**Figure 23:**
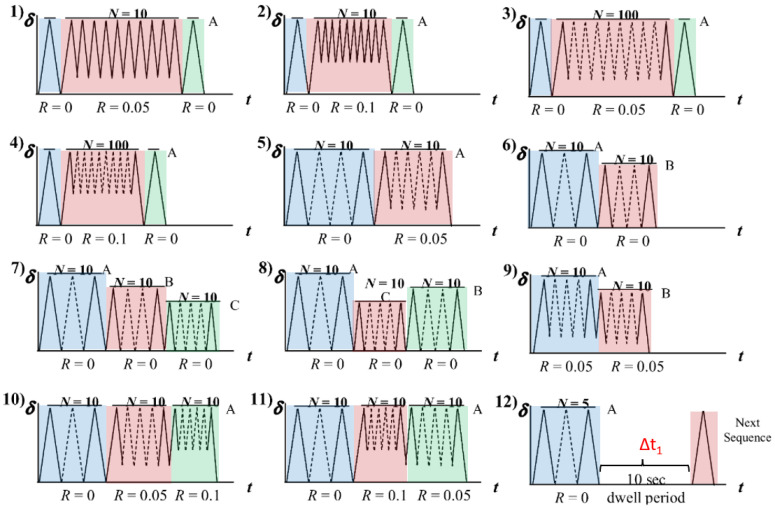
Variable amplitude loading conditions for evaluating the effects of *R*-ratios, magnitude of applied loads, sequence of loading events, and dwell periods on fatigue crack growth of filled NR and SBR materials: *δ* = displacement; *t* = time [152]. Reproduced with permission from John Wiley and Sons, 2007.

Regarding the evolution of variable amplitude loads depicted in Figure 22, Harbour et al. [152] carried out uniaxial fatigue crack growth experiments on a pure shear sample incorporating an edge notch (depicted in Section 3.2.2) under 12 types of variable amplitude loads. They incorporated a cyclic loading sequence composed of constant-amplitude loading blocks, designed to systematically examine critical parameters governing elastomeric fatigue characteristics, including *R*-ratio, magnitude of applied loads, sequence of loading events, and dwell period (see Figure 23). The blocks in their experiments contain loading paths (c) and (d), as shown in Figure 22, and the maximum engineering strain levels for NR are set to three values, namely −32.5%, −30%, and −20%, while for SBR, the three strain levels are −27.5%, −5%, and −17.5%.

**Figure 24:**
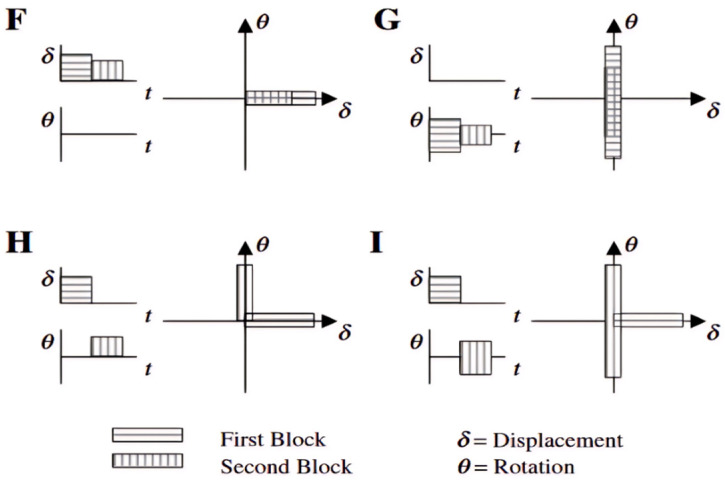
Designed loading paths for variable amplitude loadings (*δ* = displacement; *θ* = twist) [16]. Reproduced with permission from Elsevier Ltd., 2008.

The same group [16] later extended earlier works [17,152] by examining variable amplitude and multiaxial loads using the multiaxial ring test specimen (depicted in Section 3.2.1) for filled NR and SBR compounds. They carried out 14 variable amplitude tests for filled NR compound and 17 tests for filled SBR compounds, including 3 tests for load sequence and dwell period effects on fatigue life. Their variable amplitude loading paths, shown in Figure 24, can be categorized into four types: uniaxial tension blocks (*R_δ_* = 0) with amplitude levels of 7.44 and 5.00 mm, respectively (path F); pure torsion blocks (*R_θ_* = −1) with amplitude levels of 12° and 10°, respectively (path G); uniaxial tension blocks followed by pure torsion blocks (*R_δ_* = *R_θ_* = 0) with amplitude levels of 5 mm and 15°, respectively (path H); and uniaxial tension blocks followed by pure torsion blocks (*R_δ_* = 0, *R_θ_* = −1) with amplitude levels of 5 mm and 10°, respectively (path I).

**Figure 25:**
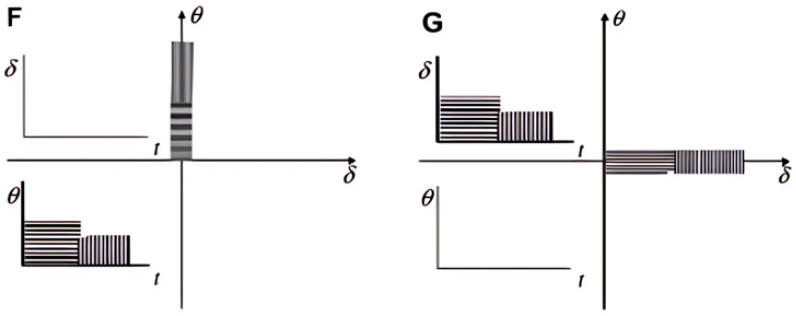
Designed loading paths for variable amplitude loadings (*δ* = displacement; *θ* = twist) [66]. Reproduced with permission from Elsevier Ltd., 2012.

To further investigate uniaxial tension blocks (*R_δ_* = 0) with various amplitude levels (like path F in Figure 24), Ayoub et al. [12] carried out 17 tests using AE42 specimens for filled SBR compound with specially designed ascending or descending amplitude levels, where the maximum axial displacement *δ_max_* falls within 7 to 22.4 mm for the two blocks. Subsequently, the same group [66] extended the various amplitude loads to pure torsion blocks (*R_θ_* = 0), as shown in Figure 25, and performed 25 tests on AE2 specimens for filled SBR compound. In their experiments, the maximum torsion displacement *θ_max_* falls within 40 to 100°for the two blocks (path F in Figure 25), and the maximum axial displacement *δ_max_* falls within 2.25 to 6.75 mm for the two blocks (path G in Figure 25).

Although there have been many contributions to fatigue tests under variable amplitude loads for rubber materials, it is evident that combined axial torsion under variable amplitude loads has not yet been thoroughly investigated. Additionally, the load *R*-ratios in published data are primarily set to 0 or −1, suggesting that more in-depth studies are needed in future research. It should be noted that the fatigue damage accumulation law under variable amplitude loading is beyond the scope of this paper. For more detailed information, readers may refer to relevant studies [1,137,158].

### 3.2. Fatigue Specimens

From an experimental perspective, some standards such as ASTM D412, D4482, and ISO 6943 [146,147,159] provide detailed guidelines for the procedures of fatigue testing and the specifications for specimen geometry. However, these standards, confining the dumbbell specimen as the only geometry of specimen for fatigue test, have been often proved inadequate for evaluating rubber components in service, particularly in scenarios involving multiaxial loads. As a result, researchers have introduced a variety of specimen geometries, which play a pivotal role in ensuring accurate interpretation of results, to accommodate the complexities of diverse testing conditions and specific experimental objectives. Following fatigue evolution progress, two types of specimen geometry used for fatigue test are available: smooth specimens and notched specimens (samples or test pieces).

#### 3.2.1. Smooth Specimens

For the fatigue testing of smooth specimens, the diversity of experiments is primarily reflected in the variety of mechanical loading modes. To investigate the fatigue properties of rubber components in service, specimens must be designed considering different types of fatigue loading, especially multiaxial loadings. Typical mechanical fatigue loadings involve various types of cyclic loading, including constant strain or stress values in simple compression or tension, shear stresses (achieved through torsional deformation or simple shear), or any combination of these.

**Figure 26:**
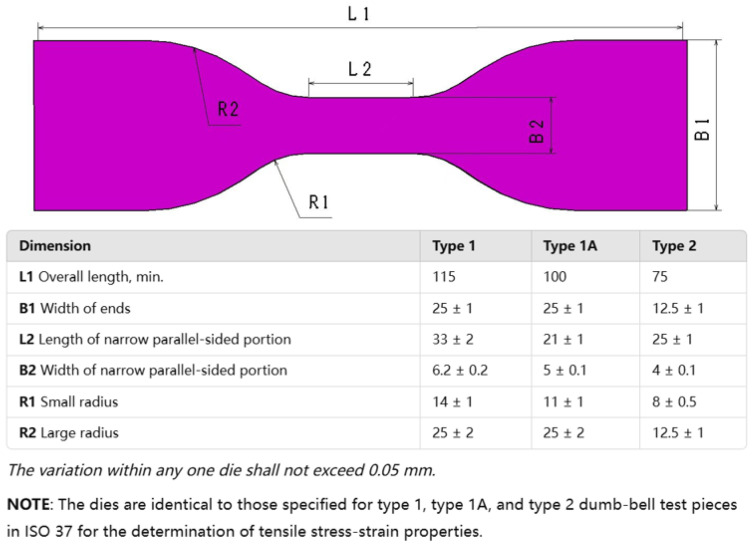
Two-dimensional dumbbell specimen suggested by standard ISO 6943 [147].

For uniaxial tension fatigue loadings, dumbbell specimens (usually taken as 2D structures, shown in Figure 26), suggested by ASTM D4482 and ISO 6943 [146,147], are the preferred choice to measure the uniaxial tension fatigue (with a loading *R*-ratio of zero or positive) of rubber, owing to its simple geometry and convenient fabrication. Also, such a specimen is frequently utilized to capture the stress–strain mechanical behavior under monotonic tension in ISO 37 [160] or limited cycles of loading and unloading tension paths.

It should be noted that certain dumbbell specimens in the literature [18] have mixed dimensions or modified dimensions from several relevant standards, such as ASTM D412, ASTM D4482, ISO 6943 and ISO 37; thus, a curvature radius was introduced to localize the deformation in the center of the specimen and consequently ensured that crack initiation will occur at this position [154]. According to standard ISO 6943 [147], the standard specimens for the determination of tension fatigue are dumbbells or rings with limited dimensions. Specimens exhibiting surface irregularities or imperfections must be excluded from testing. The reference, gage, or gauge length (distance between marked reference lines) are established as follows: 25.0 mm for Type 1 configurations and 20.0 mm for both Type 1A and Type 2 geometries. All dumbbell-shaped specimens maintain a uniform cross-sectional thickness of 1.5 mm, with a permissible tolerance of ±0.2 mm. Other dimensions are shown in Figure 26. However, the standard dumbbell specimen is unable to withstand compression or torsional loadings due to its small thickness, making it unsuitable for characterizing the compression or shear effects of rubber. As a result, some researchers have designed specimens with greater thickness to better accommodate these loadings [108,133,161]. Since there are no existing standards for fatigue testing under mechanical loading conditions other than tension, there are various names and dimensions for similar specimens used in such tests.

**Figure 27:**
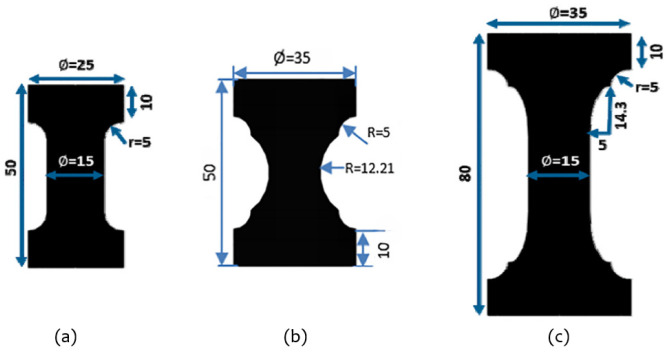
Test specimens used for fatigue life measurements: (**a**) dumbbell; (**b**) buffer; (**c**) concave [161,162]. Reproduced with permission from Elsevier Ltd., 2018.

EI Yaagoubi [161,162] successively developed three test specimens for fatigue testing (see Figure 27). The test specimen named ‘dumbbell’ is rotationally symmetric and has a free test length in the middle region, where homogeneous deformations occur, but the ‘dumbbell’ shows stress concentration in the transition area. The specimen named ‘buffer’ is also rotationally symmetric, but with a strongly inhomogeneous deformation distribution in the middle region due to the continuous cross-section changing. The specimen named ‘concave’ has two transition areas, which demonstrates that such a specimen has several advantages, for instance minimizing the stress concentrations at the transition areas, shortening the test duration, and narrowing the spread of the lifetime measurement values, compared to ‘dumbbell’ (Figure 27a) with only one transition area. Due to the elliptical transition area by the ‘concave’ specimen, there is no stress concentration near the transition area. In these works, several specimens of each geometry type were tested at different tensional load levels.

**Figure 28:**
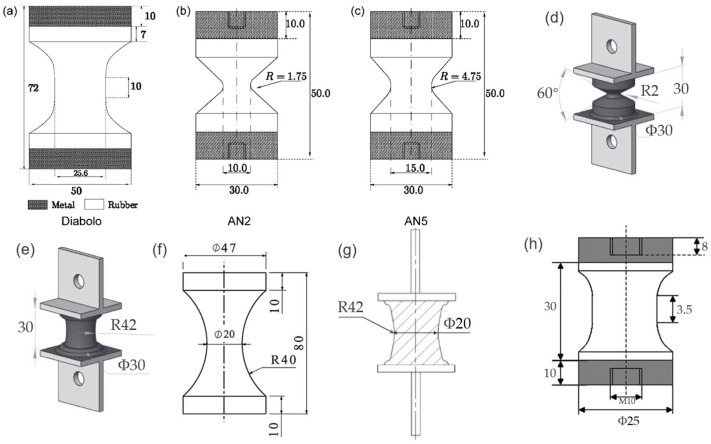
Typical specimens of SAB type: (**a**) Diabolo, (**b**) AN2, (**c**) AN5 samples [53]. Reproduced with permission from Elsevier Ltd., 2006; (**d**) AE2, (**e**) AE42 samples [154]. Reproduced with permission from Elsevier Ltd., 2023; (**f**) cylindrical dumbbell specimens [18]. Reproduced with permission from Elsevier Ltd., 2014; (**g**) specimens for tension and torsion tests [64]. Reproduced with permission from Elsevier Ltd., 2010; and (**h**) dumbbell-type specimens [36]. Reproduced with permission from Elsevier Ltd., 2011.

**Figure 29:**
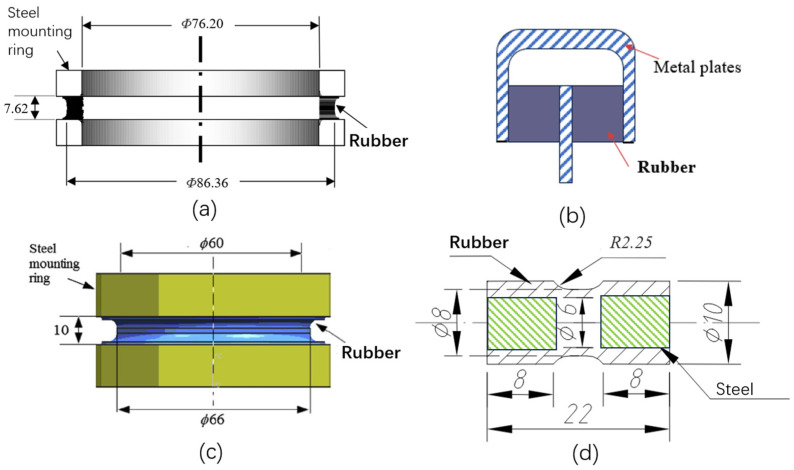
Typical specimens of HAB type: (**a**) a ring-type axial/torsion specimen [163]. Reproduced with permission from John Wiley and Sons, 2005; (**b**) a double shear unit. Redrawn by the authors based on the original data [14]; (**c**) a hollow cylindrical specimen [18]. Reproduced with permission from Elsevier Ltd., 2014; and (**d**) a hollow dumbbell specimen [156]. Reproduced with permission from John Wiley and Sons, 2008.

Axisymmetrical bodies are the preferred specimen configuration for investigating compression, shear, or torsion fatigue loadings, which can be categorized into two groups: a solid axisymmetric body (SAB), formed by rotating a 2D shape, and a hollow axisymmetric body (HAB), which is similar with SAB but with an empty interior instead of a completely filled interior. The typical SAB type include Diabolo, AN2 and AN5 specimens [53], AE2 and AE42 specimens [154], cylindrical dumbbell specimens [31], specimens for tension and torsion tests [64], and dumbbell-type specimens [36]. On the other hand, the typical HAB type include a ring-type axial/torsion specimen [163], a hollow cylindrical specimen [18] and a hollow dumbbell specimen [156]. The precise dimensions are shown in Figure 28 and Figure 29.

Such specimens (cylindrical hourglass-shaped specimens named ‘Diabolo’, AN2, and AN5) are designed to yield different local stress states under various global (or macroscopic) loadings, such as tension and compression or torsion displacements [48,53]. The specially designed ‘Diabolo’ test specimens, fabricated through molding processes, are specifically configured to achieve uniform uniaxial stress distribution during both tensile and compressive loading regimes. In contrast, the notched specimens, AN2 (featuring a 1.75 mm radius notch) and AN5 (with a 4.75 mm radius notch), are geometrically optimized to induce distinct stress triaxiality conditions, demonstrating the characteristic ratios of mean stress to equivalent von Mises stress of approximately 0.45 and 0.35, respectively, under uniaxial tensile deformation. Such three specimens were employed for fatigue characterization under ambient temperature conditions, encompassing both axial loading (tension–compression) and pure shear stress configurations, tension–compression with or without static torsion loadings. AE2 and AE42 samples are designed to carry out multiaxial fatigue tests involving different types of cyclic loading (tension, torsion, and combined tension–torsion) [154]. Particular attention should be given to the phenomenon of internal crack initiation in Diabolo specimens subjected to combined cyclic tension–compression loading with superimposed static torsional stress [48]. This internal fracture mechanism presents significant experimental challenges when investigating surface crack nucleation phenomena, particularly considering that fatigue life assessment typically employs the formation of visible surface cracks measuring approximately 1 mm in length as the failure criterion [154].

The fabrication of appropriate rubber specimens is a critical issue that requires careful consideration to achieve specific experimental objectives. In general, specimen design needs to fulfill the following three requirements: to generate a homogeneous strain distribution throughout the gauge section, to assure preferential surface crack initiation to enable optical monitoring of crack growth and to permit structural stability under compressive loading to prevent buckling or wrinkling, as well as to be useful for characterizing the stress–strain behavior of rubber [163]. Additionally, the specimen geometry must be easy to mold, compatible with typical axial–torsion load cells, capable of large axial and shear strains within common machine strokes and frequencies and prevent debonding from the mounting metal parts.

The HAB specimens might be prone to initiate preferential surface cracks because of the intermediate thickness to facilitate enhanced optical accessibility for crack propagation monitoring. According to the requirements of a proper specimen mentioned above, Mars and Fatemi [163] proposed a ring-type axial/torsion specimen. It has been reported that such a specimen can withstand combined engineering shear and axial strains, with axial strain ranging from −30% to 200% and shear strain from −250% to 250%. The peak principal strain spans 0 to 200%, with the stretch biaxiality ratio B—mathematically expressed as the logarithmic ratio of transverse principal stretch to peak principal stretch—and maintains values of within −1 to 0. The range of stretch biaxiality ratio can be expanded into positive values through introducing internal or external pressurization in the gage section or potentially using a modified specimen with an extended gage length. The synergistic application of axial and pressure loading can cover the full range of −1 < *B* < 1. However, the compressive deformation capacity of this specimen configuration is inherently constrained, with maximum achievable axial compressive strains typically limited to approximately 30% due to the onset of surface instability phenomena characterized by localized wrinkling patterns.

Similarly, Wang et al. [156] designed a hollow dumbbell specimen specifically engineered for multiaxial fatigue characterization, incorporating a reduced gauge section to prevent torsional instability during testing. Thereafter, Shangguan et al. [18] developed a scaled-down version of ring-type axial/torsion specimen proposed by Mars and Fatemi [163].

For all the specimens, the elastomeric parts were bonded to metallic parts through in situ vulcanization, achieving interfacial adhesion during the curing cycle. Generally, they have a larger radius at both ends and a smaller radius at the center to ensure that the crack initiation occurs at the center ozone. Finally, it should be noted that many tests were carried out three times and the mean fatigue life is an average value of the three tests [154,164]. However, according to ASTM D4482 [146], six specimens should give a representative measure of the median for NR, but for SBR and rubbers that behave similarly, 12 test specimens are likely to be required. It is worth nothing that more repeated tests are required to achieve a better statics data.

#### 3.2.2. Notched Specimens

Notched specimens are also known as crack growth test specimens. The shape of such specimens directly influences the formula used to calculate the tearing energy. The specimen’s geometry affects stress distribution and deformation patterns around the crack tip, which in turn impact the derivation of the tearing energy formula. The choice of specimen shape also determines the applicability of specific fracture mechanics models, such as linear elastic fracture mechanics (LEFMs) or nonlinear elastoplastic approaches. For example, compact tension specimens [165] are common for mode I/II fracture tests, while cruciform or ring-shaped specimens are better suited for mixed-mode or multiaxial testing. Therefore, when designing crack propagation experiments, the careful consideration of specimen shape is essential to ensuring that the resulting tearing energy calculations accurately reflects the material’s fracture behavior under the tested conditions.

There are some standards, e.g., ASTM D813, ASTM D430, and ISO 132 [166,167,168], that recommend testing requirements to obtain the flexing fatigue of rubber materials. However, the majority of the literature on rubber fatigue is focused on tensile or shear fatigue, since many rubber products, such as seals, tires, isolators, and conveyor belts, predominantly experience tensile or shear loads during operation, leading to relatively higher research demand in this area. Furthermore, flexing fatigue can be regarded as a combination of tensile and shear fatigue, and to some extent, its behavior can be indirectly explained using existing tensile or shear fatigue models.

In this respect, various notched configurations under tensile loading conditions have been extensively employed in fracture mechanics research to characterize the critical tearing energy of elastomeric materials. These include single-edge notched tension (SENT), double-edge notched tension (DENT), center-cracked tension (CCT), and pure shear (PS) specimen geometries, as documented in [169]. Of these, SENT and PS geometries (shown in Table 4) are more commonly used due to their simpler preparation methods. Some researchers also name them single–edge–cut or edge-cracked simple tension samples and single–edge–cut planar tension specimens, respectively. To avoid confusion, these geometry types will be referred to as SENT and PS specimens in the present paper. Additionally, the trouser tear specimen (see Table 4) was extensively used in earlier studies [32] on fatigue crack growth in rubber materials, primarily to establish the geometric independence of the correlation between the energy release rate and the fatigue crack growth rate.

The SENT specimen is typically employed for tearing energies near the threshold of tearing energy *T*_0_; otherwise, the PS specimen with an edge cut is frequently utilized for fatigue crack growth during other phases [112,173]. As for SENT specimen geometry, the crack length *a* must be small compared to the width *w* to assure that the crack front remains far enough from the specimen’s opposing boundary to eliminate edge interaction effects, so that there is a uniform strain energy density at the free edge as if the crack were not present. The formula of tearing energy for SENT specimens is presented in Table 4.

The PS specimen geometry incorporates a high width-to-height ratio, effectively restricting transverse deformation through grip constraints while facilitating uniaxial loading along the reduced dimension. The term ‘pure shear’ refers to the fact that, for moderate and incompressible strain levels, this strain state corresponds to pure shear when expressed within a suitably rotated coordinate system relative to the specimen. Furthermore, for such a specimen, the formula for tearing energy *T* has an especially simple form (shown in Table 4), assuming that the cut is sufficiently deep and the crack tip fields translate without altering their configuration during crack growth. The formula for the PS specimen has the same limitation as SENT specimen, as *W* must be determined indirectly. Pure shear geometry takes the advantage that the ERR is independent of the crack length so that crack growth rate is not intensified by increasing crack length and that the assessment of fatigue crack growth occurs under steady conditions [169].

Unlike the analogous testing of metals (for example, the trouser tear specimen would produce a mode III crack in a metal), the three specimens in Table 4 produce similar values of tearing energy *T* at the fracture and generate mode I (opening) cracks, since the crack tip is perpendicular to the tension, owing to the buckling of rubber during extension [32]. Therefore, many researchers prefer to employ the PS specimen to test fatigue properties of rubber in their works [106].

## 4. Reinforcement Methods for Rubber-Based Structures Against Fatigue Failure

Of four types of factors affecting elastomer fatigue, in-service loading histories and environmental conditions of a real rubber component are difficult to alter, except through the implementation of certain isolation measures [3]. Therefore, efforts should be directed towards the latter two factors—rubber formulation and the characteristics inherent to the constitutive behavior of rubber—to improve fatigue life of elastomeric components.

Tavosi et al. [106] suggested that two approaches can improve the toughness and the resistance against the crack propagation of rubber-like materials: one is employing reinforcing fillers and the other is using polymeric resins that tend to increase the viscoelastic dissipation and the tear strength.

The fatigue-induced failure mechanism encompasses both the nucleation of microscopic crack precursors and the subsequent growth of inherent material discontinuities. These defects likely originate from multiple contributing factors, including fillers, foreign matter contamination, matrix porosity, the non-uniform distribution of formulation components, and surface irregularities [3,8]. Therefore, in terms of material compounds, rubber fatigue behavior is influenced by factors such as polymer matrix composition, the type and volume fraction of fillers, and processing conditions. Additionally, formulation additives, particularly stabilizers (antioxidants and antiozonants) and crosslinking agents, play a significant role in fatigue performance. A critical factor is whether the compound exhibits strain crystallization, as strain-crystallizing rubber materials are less susceptible to environmental impacts [7]. Carbon black (CB) improves fatigue resistance characteristics, with low-structure carbon blacks offering enhanced performance compared to high-structure counterparts [7,174]. There are a large number of research studies in materials and material processing fields [175,176] for improving the fatigue resistance of rubber, but this section focuses on utilizing crystallization and reinforcement fillers to enhance rubber fatigue performances.

### 4.1. Crystallization

Some polymers contain, in their molecular structure, a regularity which allows the chains to group in small regions of local order. These regions are called crystallites. Polymers with high crystalline contents behave as a plastic (e.g., polyethylene) and those with a low crystalline content behave as an elastomer (e.g., rubber) [121]. The presence of crystallites strongly influences the viscoelastic behavior of the polymers. In particular, Young’s modulus and the tear strength of crystalline polymers are higher than other purely amorphous polymers, at the same relative temperature above their glass transition temperature. If crystallization is prevented in these materials, their strength behavior is the same as for other purely amorphous polymers.

Natural rubber (NR), a typical rubber material that undergoes strain-induced crystallization, is often the focus of research aimed at quantifying fatigue resistance behavior [150]. Such a material is purely amorphous when it is in the unstrained state. However, when subjected to substantial tensile deformation under ambient conditions, NR undergoes a strain-induced crystallization transition, resulting in the formation of a semicrystalline-like structural configuration, characterized by highly aligned microstructure domains oriented parallel to the applied tensile direction [121]. The degree of crystallinity increases with increasing strain, and for NR, it would reach a maximum value of around 30% [20]. The crystallites also add to the tear energy by requiring energy for their formation, which is then not available for the crack to propagate. When the cyclic loading conditions maintain the crack–tip tensile stress above the critical threshold for crystallization initiation, the material sustains a persistent crystalline phase, effectively inhibiting crack propagation through enhanced mechanical resistance.

### 4.2. Reinforcing Fillers

When a rubber component with fillers is subjected to dynamic loading conditions, the rubber matrix surrounding the filler experiences greater strain compared to the rest of the matrix. Cyclic mechanical loading can induce interfacial debonding at the filler–matrix boundary, initiating microcrack nucleation. Consequently, the interfacial adhesion strength between reinforcing particles and elastomeric matrix and the dispersion level of fillers play a critical role in microcrack formation. Additionally, crack propagation is influenced by the material’s tearing energy, with well-dispersed filler particles acting as effective obstacles to crack propagation [177].

The strength of purely amorphous elastomers can be enhanced by the addition of reinforcing agents. The most common reinforcing agent in use is fine particles of carbon black (CB), used in relatively large amounts (approximately 50% by weight of the elastomer). The enhancement of tear strength can be substantial (an order of magnitude), but the reinforcement is limited to a narrow range of tear rates and test temperatures. In the rate and temperature range of reinforcement, the tear does not grow smoothly through the elastomer as it does for a non-crystallizing elastomer, but progresses through a discontinuous stick-slip process [20]. Kim and Jeong [178] experimentally examined the influence of carbon black (CB) on fatigue resistance through three types of filled NR compounds, N330, N650, and N990 (the small-, medium-, and large-sized CB) on hourglass-shaped fatigue specimens, and found that the fatigue life of N650 is shorter than that of N330 and N990, which indicates that existence of large CB agglomerates positively or negatively influence the fatigue life of NR.

Nano-clay, specifically montmorillonite [179], is widely used as a reinforcing filler to improve the fatigue resistance of rubber materials and components [180]. The advantages of nano-clay over other fillers have also been explored from various perspectives [181]. A study conducted by Wu et al. [177] investigated the flexural fatigue resistance of SBR composites containing dual fillers of clay and carbon black (CB). The experimental results demonstrated that the addition of 4–5 phr (parts per hundred rubber) of nano-dispersed clay significantly enhanced the fatigue life of the composites. Contrary to conventional expectations, the inclusion of clay particles maintained the crosslinking density while simultaneously enhancing both hysteresis properties and tear resistance. The environmental scanning electron microscopy analysis of fracture surfaces under flexural conditions revealed that nano-dispersed clay layers exhibited superior crack-arresting capabilities compared to conventional carbon black (CB) fillers, primarily through a crack-blunting mechanism. Bakhshizade et al. [182] investigated a blend of NR and SBR with and without nanoclay particles. However, they found that at high-strain amplitude values, increasing the amount of nanoclay from 5 to 7 phr in the rubber composition did not result in a significant improvement in fatigue life and, in some cases, led to a reduction in performance. Izadi et al. [179] considered incorporating small amounts of nanoclay into a nitrile butadine rubber (NBR) matrix, and they observed the life of NBR/nanoclay composite was enhanced compared to neat NBR. Rooj et al. [183] experimentally evaluated the influence of expanded organo-montmorillonite (EOMt) nanoparticles on the microstructural characteristics and fracture mechanics of carbon black (CB)-filled NR composites. The findings indicate that the incorporation of a relatively low concentration (5 phr) of EOMt nanoparticles resulted in a substantial decrease in the crack propagation rate within the composite material. The nanocomposite design of elastomers is an important element for improving the service life of rubber components [179].

Currently, numerous studies are being conducted to explore reinforcing fillers to enhance the fatigue resistances of rubber materials. For instance, Liang et al. [184] studied the effect of hemp fiber (HF) length variations on the mechanical and fatigue properties of styrene-butadiene rubber/carbon black (SBR/CB) composites.

## 5. Recent Progress on Fatigue Analysis of Magnetorheological Elastomers (MREs)

Magnetorheological elastomers (MREs), consisting of micron-scale ferromagnetic particles embedded in a non-magnetic polymer matrix, are advanced rubber-like materials that have attracted global interest due to their tunable viscoelastic properties (stiffness and damping) under external magnetic fields [2], which is known as the magnetorheological (MR) effect. MREs can be effectively used to design adaptive absorbers and isolators to attenuate vibration under a wide range of frequencies [185]. They may be subjected to various loading conditions, including shear, compression, tension, and multiaxial loads, under zero-field and applied magnetic field conditions. Additionally, their long-term mechanical properties, such as fatigue behavior, are critical for ensuring the durability of MRE-based devices for long-term applications.

**Figure 30:**
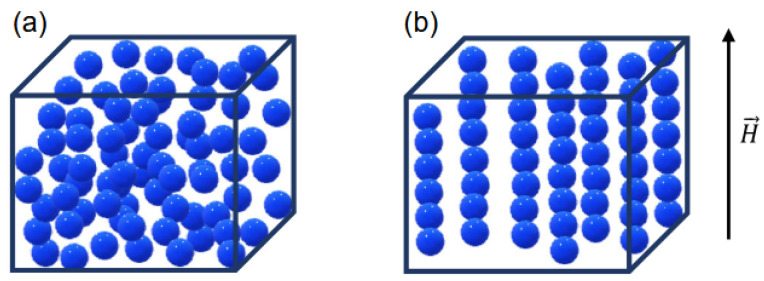
Schematic diagram of (**a**) isotropic MRE and (**b**) anisotropic MRE structures [2]. Reproduced with permission from John Wiley and Sons, 2023.

In general, MREs can be classified into two types, namely isotropic and anisotropic, based on significant differences arising from the fabrication process with or without strong external magnetic fields. In isotropic MREs, magnetic particles are randomly dispersed in the elastomeric matrix during the fabrication process without magnetic fields. On the other hand, in anisotropic MREs, magnetic particles form a chain-like distribution during fabrication with magnetic fields, as shown in Figure 30. Some studies [186] proved that anisotropic MREs appear to be more sensitive to applied magnetic fields with a slightly larger MR effect than isotropic MREs in most scenarios.

The fatigue behavior of MREs is affected by various material and loading parameters, similar to factors influencing rubber fatigue. Key material factors include the type of matrix and magnetic particles, particle concentration, and the presence of additives. Mechanical and magnetic loading conditions, such as frequency, amplitude, temperature, and magnetic flux density, also play a significant role. Understanding these influences on the fatigue behavior of MREs is essential for designing and optimizing MRE-based adaptive structures and systems. Research on MRE fatigue remains limited, highlighting the need for a better understanding of fatigue prediction [187].

As discussed in Section 1, Section 2, Section 3 and Section 4, research on rubber fatigue has expanded considerably, with almost 400 studies published since 2002, underscoring its significance in the rubber industry. In contrast, research on MRE fatigue is still scarce, emphasizing the need for the improved understanding and prediction of fatigue behavior [187]. Currently, there are no standardized fabrication specifications, fatigue testing or specimen geometries for MRE fatigue characterization. The best practice is to apply test standards for rubber into MRE due to considerable similarity, especially in the matrix system [187]. Instead, researchers designed specimens and fatigue tests to suit the complexity of testing conditions.

**Figure 31:**
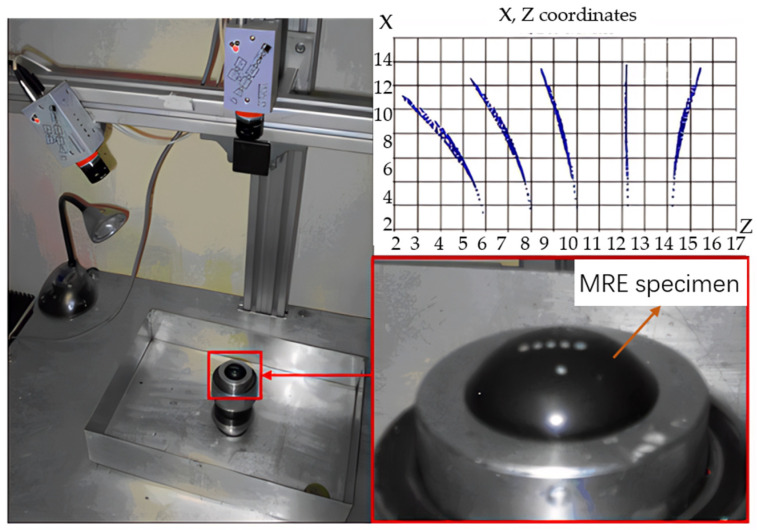
The equi-biaxial dynamic bubble inflation system [188]. Reproduced with permission from Elsevier Ltd., 2014.

One of the first experimental works on the fatigue properties of MREs was written by Zhou et al. [164]. They developed a hydraulic-based bubble inflation apparatus integrated with an optical measurement and real-time data acquisition system [188], as shown in Figure 31. The specimen is designed as a disk shape with a 49 mm nominal diameter and a 1 mm thickness. The engineering stress ($\sigma_{E}$) and principal stretch ratio (λ) at the bubble pole are attained as follows [189]:

(15) $$\sigma_{E}=\lambda p\left(r/2t_{0}\right)$$

(16) $$\lambda =\left(X_{c}-X_{o}\right)/X_{o}+1$$

where p (MPa) denotes the inflation pressure; *r* (mm) indicates the curvature radius; $t_{0}$ (mm) represents the initial specimen thickness; $X_{c}$ signifies the circumferential point spacing at the pole; and $X_{o}$ corresponds to the initial reference spacing.

The studied MR material consists of 20% carbonyl iron powder (CIP) distributed in silicone rubber. The stress and strain at failure, as well as the cycles to fatigue failure under four levels of stress amplitude, were measured during the equi-biaxial fatigue tests. It was found that stress softening occurred and there was a limit to the number of complex moduli corresponding to the fatigue failure of MRE, irrespective of stress amplitude and loading method.

The same research group [188,190] further explored the behavior of MREs by varying the concentration of CIPs and comparing isotropic and anisotropic MREs. They concluded that dynamic stored energy (SED, calculated using the unloading stress–strain curve) is a more reliable fatigue damage parameter than maximum stress, strain, total energy, or dissipated energy when predicting the fatigue life of silicone-based MREs under equi-biaxial loadings. This conclusion was obtained from their observation of dynamic stored energy evolution over cycles, where they observed a consistent trend of increasing stored energy density with progressive cycling, independent of both applied stress levels and particle content. Notably, the stored energy density at failure appeared to approach a limiting value. It should be noted that this investigation represents an initial exploration, as all cyclic testing was conducted in zero-field conditions without magnetic field applications. Additionally, the fatigue loading *R*-ratio was zero, and there was no comparison between equi-biaxial and uniaxial fatigue data to determine whether SED or other damage parameters were able to unify the results.

Zhou et al. [191] extended the equi-biaxial fatigue research for MREs with different CIP contents into the situation under external magnetic fields (uniform mean magnetic field strength of 400 mT). They found the fatigue life, the complex modulus, the damping loss factor is increased by different levels under external magnetic fields. The stress–strain property is highly influenced by magnetic fields since increased interactions between CIPs restrict the mobility of elastomer chains. MREs still exhibit a critical complex modulus (E*) at failure, with maximum stress and stored energy serving as effective fatigue life predictors under external magnetic fields. One shared characteristic in the aforementioned research studies by Zhou et al. [164,188,190,192] is that all damage parameters were obtained from basic stress–strain test data, and no finite element modeling or calculation was considered. This indicates that the complex constitutive behaviors of MREs were not taken into account and that the results are limited to the specimen level.

To detect the MREs’ micro-damage and predict the service life in the future, some researchers [193,194,195,196,197,198] have devoted significant efforts to studying the evolution of mechanical properties of MREs under cyclic loading. Gorman et al. [193,194] investigated the evolution of MR effect in NR-based MREs with magnetic field strengths, strain limits, and strain amplitudes during both uniaxial and biaxial cyclic tests. They observed that in both series of tests, the largest MR effect was observed for low strain levels, while the lowest MR effect occurs in fatigue loadings with low strain amplitude and a high upper-strain limit. Additionally, the modulus increased with an increasing magnetic field, which is a consistent experimental trend, as seen in the other experimental studies. Wang et al. [196] explored the relationship between impedance and the dynamic mechanical properties of NR-based anisotropic MREs under fatigue loading, with the aim that the non-destructive impedance spectrum method has the protentional ability to detect the damage of the MRE microstructure in real-time and predict the fatigue life in practical application. Recently, Lian et al. [195] investigated the magnetic fatigue properties of a silicone rubber-based MRE under repeated external magnetic field conditions. They found that the shear moduli decreased when increasing the number of fatigue cycles in both cases—with and without a magnetic field—and that the hysteresis loss under magnetic field conditions is smaller than that under no magnetic field conditions. Additionally, the MRE became softer due to CIPs undergoing slight movement as the number of cycles of the repeated application of a magnetic field increased. Their findings contribute to understanding the fatigue mechanisms of MREs, particularly under external magnetic fields; however, they did not focus on the prediction methods of fatigue life of such an MRE in uniaxial or biaxial cyclic fatigue tests.

**Figure 32:**
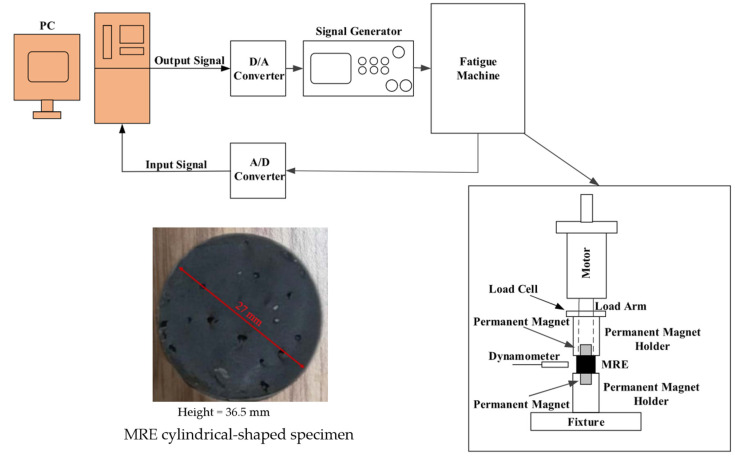
MRE specimens and multi-axial fatigue testing machine [200]. Licensed under a Creative Commons Attribution License (CC BY 4.0).

To explore complex constitutive behaviors of silicone rubber-based MREs during fatigue life prediction, very recently, Googarchin et al. [199] proposed a novel 3D magneto-hyper-viscoelastic semi-coupling constitutive model, and employed SED as a fatigue damage parameter for assessing multiaxial fatigue performance in MREs with varying CIP volumes under different external magnetic fields. They designed a cylindrical-shape MRE specimen with a diameter of 27 mm and a height of 36.5 mm and developed a multi-axial (specially combined tension–torsion) fatigue test machine (see Figure 32).

They found that the Mooney–Rivlin [201] SED fatigue damage parameter (where the hyper-elastic parts of the MRE’s magneto-hyper-viscoelastic properties are characterized using the Mooney–Rivlin model, as referenced hereafter) provided the most accurate results across all CIP contents. The Yeoh [202] SED predictor performed better for MREs with higher CIP contents, while the Neo-Hookean [203] SED predictor was found to be inadequate for any CIP content. It should be noted that the cyclic loadings applied to the MRE cylindrical specimen in the multi-axial fatigue tests were combinations of tension (with values of 2, 5, 10, 15, and 20 N) and torsion (with corresponding torsion torques of 2, 5, 10, 15, and 20 N·mm). These loadings had a mean of zero tension force and zero torsion torque and were applied with or without a constant magnetic field (0.27 T or 0.4 T) generated by two neodymium magnets (N52).

Meanwhile, Hosseini et al. [204] expanded upon the work of Googarchin et al. [199] by investigating the fatigue properties of MREs under repetitive magnetic fields and cyclic compression/tension loading conditions. They fabricated silicon rubber-based isotropic MREs with a fixed volume fraction of 20% CIPs and subjected the samples to multi-axial fatigue testing with a variable magnetic field generated by solenoids. They found that the maximum stress could serve as a reliable fatigue damage parameter for MREs under the combination of repetitive magnetic and cyclic loading. In terms of mechanical loadings, the magnetic and cyclic forces were in-phase—when the cyclic forces reached their maximum value, the magnetic force also reached its peak (400 mT). Their findings provide valuable insights into developing effective fatigue evaluation methods for MREs. However, further research on more complex fatigue loading conditions, such as non-proportional loadings, is needed to assess the effectiveness of SED or maximum stress as damage parameters for predicting the fatigue life of MREs under real-world service conditions.

Additionally, the matrix materials in the aforementioned fatigue-related research studies on MREs are limited to silicone rubber (SR) and natural rubber (NR), while the magnetic particles used are CIP. The rubber compound in MREs’ fatigue studies are less diverse than those used in rubber fatigue studies [10] and those used in mechanical modeling of MREs [2].

Last but not least, the abovementioned MRE fatigue research studies lack a comprehensive consideration of complicated nonlinear constitutive behaviors, such as hyper-elastic, magneto-elastic, and viscoelastic properties. An accurate material constitutive model to capture the nonlinear magneto-mechanical behavior of MREs is a prerequisite for estimating damage parameters to predict fatigue life, especially for complex MRE-based structures under multiaxial loadings. With regard to constitutive equation of MREs, typical approaches include macro-continuum-based models, microparticle interaction-based models, and data-driven phenomenological models [2]. The continuum-based approach treats the MREs as equivalent homogeneous composites to study their macroscopic response while neglecting microstructural effects. The microstructure-based models generally consider the interaction between micrometer-sized magnetic particles within the elastomeric medium considering different lattice configurations. The seminal models, which consider only the magnetic interaction between adjacent point dipoles, offer the advantage of understanding the underlying microstructural magnetic behavior without directly addressing the complexities of coupled magnetic and mechanical fields. Data-driven phenomenological models mainly characterize the dynamic and viscoelastic behaviors of MREs through extensive experimental studies for both isotropic and anisotropic soft MREs, apart from the hyper-elasticity, which is focused on by the first two approaches. More descriptions are provided in the literature review [2].

## 6. Discussions and Conclusions

The present paper attempted to collect and analyze recent works on rubber fatigue in the literature in the past decade with the main objective to enhance current reviews by incorporating recent advancements and previously overlooked research developments.

Five analysis approaches for the evaluation of fatigue life in rubber materials were described: the crack nucleation approach, the crack propagation approach, the fatigue phase-field method, the probabilistic fatigue method, and the machine learning (ML)-based method. Of these, the latter two approaches are based on the damage parameters of the crack nucleation approach. In the crack nucleation approach, a number of damage parameters were proposed to unify various fatigue data under different mechanical loads. However, existing approaches appear to be limited in effectiveness, especially in terms of unifying multiaxial and uniaxial loads with different loading ratios. Researchers investigating rubber fatigue typically focus on specific aspects, which provides insight into certain facets of rubber fatigue. However, a comprehensive understanding of its full nature will require broader, interdisciplinary, and collaborative efforts across various fields of study. In the crack propagation approach, a key challenge lies in accurately computing crack-specific energy release rates with high efficiency. Regarding the fatigue phase-field method, a potential evaluation of a broader range of loading conditions and substantial computational resources is required, which delays their applications in rubber components. As for the probabilistic fatigue approach, it benefits the explanation of data scattering of fatigue life and also relies on the proper damage parameter. As learning algorithms continue to develop and diversify, more machine learning (ML)-based studies are expected to emerge for predicting rubber fatigue lifespan. The ML-based approach is particularly useful for expanding datasets, especially when dealing with small samples of data. However, it relies on the selection of suitable damage parameters and cannot fully reveal the underlying fatigue damage mechanisms of rubber.

Building on the analysis approaches mentioned previously, typical fatigue experimental results from the literature are gathered and examined based on mechanical loading types and fatigue specimens. This paper identifies a gap in experimental studies, highlighting the lack of data on non-relaxing shear loads, combined axial torsion loads for different rubber compounds, and combined axial torsion paths under variable amplitude loads. While some experimental data on the strain crystallization effect, especially under tension–tension loads, are available for certain rubber compounds, a unified model able to accurately quantify this effect of fatigue on the lifespan of rubber is still in progress.

Reinforcement methods for rubber materials and components against fatigue failure were subsequently discussed, focusing on crystallization and reinforcing fillers, as extending fatigue life or improving fatigue resistance is the ultimate goal of many fatigue-related studies. A significant amount of research is being conducted to explore reinforcing fillers to enhance the fatigue resistance of rubber materials. Additionally, many studies focus on the fatigue performance of rubber components. However, there is a lack of connection between the material level and component level, which paves the way for the future multiscale modeling of rubber fatigue.

Finally, recent progress in the fatigue analysis of MREs is reviewed. The fatigue analysis of MRE is in its infancy and substantial research is required to understand the fatigue behavior of MREs under the simultaneous applications of non-proportional mechanical and magnetic loadings. Complicated nonlinear constitutive behaviors, such as hyperelastic, magnetoelastic, and viscoelastic properties, need to be considered in fatigue analysis.

It should also be emphasized that research focused on rubber fatigue under high temperatures or thermal cycling, a topic that has garnered considerable attention over the past decade [205,206,207,208,209,210], has been excluded from the scope of this review study due to space and length constraints. However, given the growing interest in these areas, such research could be a valuable subject for a more detailed review in the future, considering its relevance to understanding the long-term performance and durability of rubber materials under varying environmental conditions.

## Figures and Tables

**Table 1:** Summary of characteristics of multiaxial loads.

Type of Loading	Stress/Strain Magnitude	Timing	Principal Directions
Equi-biaxial	Equal stress/strain in two directions.	Simultaneous (no phase shift)	Constant, aligned with load axes.
Inequi-biaxial	Unequal stress/strain in two directions.	Simultaneous (no phase shift)	Constant, aligned with load axes.
In-phase	Any stress/strain ratio (equal or unequal).	Synchronized (no phase shift)	Constant throughout the cycle.
Out-of-phase	Any stress/strain ratio (equal or unequal).	Asynchronous (with phase shift)	Rotates during the cycle.
Proportional	Constant stress/strain ratio in all directions.	Either in-phase or out-of-phase	Constant throughout the cycle.
Non-proportional	Variable stress/strain ratio in all directions.	Either in-phase or out-of-phase	Rotates during the cycle.

**Table 2:** Summary for test data under pure torsion. Created by the authors using original data [17].

Path Code	Number of Test Data	The Twist Range	Objective of the Designed Path
B	14	6°~23.2° (*θ_max_*, *R_θ_* = 0)	Path B and C are for crack closure effect.
C	9	±5.4°~±15° (*R_θ_* = −1)	
J	2	±9°, −14.8°~14.6° (*R_θ_* ≈ −1)	Path J, K, and M are for the effect of static axial load on rubber fatigue.
K	2	±8°, ±12° (*R_θ_* = −1)	
M	4	±7°, ±7°, ±12°, ±15° (*R_θ_* = −1)	

**Table 3:** Elaborations for each combined axial torsion loads. Created by the authors by analyzing original data in Mars and Fatemi [17].

Path Code	Number of Test Data	Global Loads		Description
		Displacement Peak *δ_max_* (mm)	Twist Peak *θ_max_* (°)	
D	16	0.76~5.01 (*R_δ_* = 0)	3~20 (*R_θ_* = 0)	Proportional tension–torsion loading (*R_δ_* = *R_θ_* = 0), where paths D_1_ and D_2_ with different ratios of axial strain to shear strain (0.25 and 0.5, respectively) are used to identify the effect of proportional loading
E	5	0.75~2.03 (*R_δ_* = −1)	3~8 (*R_θ_* = −1)	Proportional tension–torsion loading *(R_δ_* = *R_θ_* = −1)
F	3	−1.51 (*R_δ_* = ∞)	12~20 (*R_θ_* = 0)	Proportional compression-torsion loading
G	3	2~3.58 (*R_δ_* = 0)	7~14 (*R_θ_* = 0)	Non-proportional tension–torsion loading (phase angle *φ* = 45°) (*R_δ_* = *R_θ_* = 0)
H	5	0.76~3.53 (*R_δ_* = 0)	3~14.1 (*R_θ_* = 0)	Non-proportional tension–torsion loading (phase angle *φ* = 90°) (*R_δ_* = *R_θ_* = 0)
I	4	0.76~3.53 (*R_δ_* = 0)	3~14.1 (*R_θ_* = 0)	Non-proportional tension–torsion loading (phase angle *φ* = 180°) with maximum strain rotation (*R_δ_* = *R_θ_* = 0)
L	5	−0.76, −1.52 (*R_δ_* = ∞)	10~20 (*R_θ_* = 0)	Non-proportional compression–torsion (phase angle *φ* = 180°)
N	2	1.36, 3.13 (*R_δ_* = 0)	5.9, 13.6 (*R_θ_* = 0)	Proportional tension–torsion loading (load control) (*R_δ_* = *R_θ_* = 0)
O	2	1.33, 4.27 (*R_δ_* = 0)	5.4, 13.2 (*R_θ_* = 0)	Non-proportional tension–torsion loading (load control) (*R_δ_* = *R_θ_* = 0)

**Table 4:** Typical notched specimens and formulas to estimate tearing energy (TE).

Specimen and Formula for TE	Notched Specimen Shape and Loading	Illustrations [169]
Edge crack panel or single-edged notched tension (SENT) T=2kWa	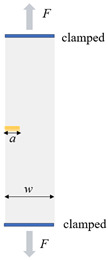	Where k is determined empirically and $k=\frac{\pi }{\sqrt{\lambda }}$ [170] or $k=\frac{2.95-0.08(\lambda -1)}{\sqrt{\lambda }}$ [171] are usually used. λ is the extension ratio; *W* is the SED as if there were no crack. The SED is not uniform within the specimen so it should be obtained via measured stress–strain data from another similar test on an uncut specimen or through finite element analysis.
Planar tension or pure shear specimen (PS) $T=Wh_{0}$	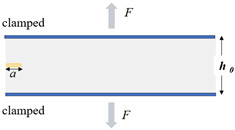	Where *h*_0_ denotes undeformed height of the specimen and *W* is strain energy density as if there were no crack.
Trouser tear piece (ASTM D624-Type T) [172] $T=\left(2\lambda F-WA_{0}\right)/t$	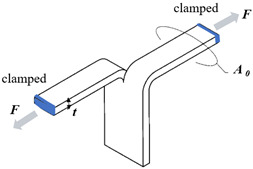	Where λ is the extension ratio; *A*_0_ is the cross-section area of an undeformed single leg; *F* is the external tensional load on the leg; and *W* denotes strain energy density.

## Data Availability

This is a review paper and all data supporting the findings of this study are included within the manuscript.

## Symbols and Abbreviations

The following symbols and abbreviations are used in this manuscript:

λ, $\lambda_{m}$, and $\lambda_{a}$	stretch ratio (elongation), mean, and amplitude, respectively;
$\varepsilon_{E}$	engineering (nominal) strain
$\varepsilon_{G-L}$	Green–Lagrange strain,
$\varepsilon_{A-E}$	Almansi–Euler strain
$\varepsilon_{L}$	logarithmic strain
$\varepsilon_{o}$	octahedral shear strain
$E_{ij}\;(i,j=1,2,3)$	strain components of Green–Lagrange strain tensor E
$\varepsilon_{f}$	effective tensile strain criterion
$\varepsilon_{fi}(i=1,2,3)$	principal components of $\varepsilon_{f}$
$\sigma_{\max}$	maximum eigenvalue of the Cauchy stress tensor
d*a*/d*N*	crack growth rate where the crack length is *a* and the number of cycles is *N*
*T*_max_	the maximum tearing energy
*U*	internal energy stored in the component
*A*	crack area of a single fracture surface
W and $W_{c}$	SED and CED, respectively
*R*-ratio (*R**_γ_***, *R**_ε_***, *R_δ_ or R_θ_*)	ratio of minimum to maximum load during one cycle
*δ* and *θ*	displacement and twist/rotation, respectively
*P* and *T*	axial load and torque, respectively
γ, $\gamma_{m}$, and $\gamma_{a}$	shear strain (the twist per unit length), mean value, and amplitude, respectively
ω	the signal frequency
φ	the phase angle between extension and twist
*B*	stretch biaxiality ratio (defined as the ratio of log of transverse principal stretch to log of maximum principal stretch)
*S*–*N*	stress versus number of cycles to failure
*E**	complex modulus
CB	carbon black
HF	hemp fiber
MREs	magnetorheological elastomers
MR	magnetorheological
EOMt	expanded organo-montmorillonite
SR	silicone rubber
NR	natural rubber
SBR	styrene butadiene rubber
SED	strain energy density
CED	cracking energy density
EPDM	ethylene propylene diene monomer rubber
FEA	finite element analysis
ERR	energy release rate
TE	tearing energy
SWT	Smith–Watson–Topper
CXH	Chen–Xu–Huang
CMA	configurational mechanics approach
CPA	critical plane approach
CLD	constant life diagram
1D, 3D, 6D	one-, three-, six-dimensional
CDM	continuous damage mechanics
SENT	single-edged notched tension specimen
LEFM	linear elastic fracture mechanics
MCS	Monte Carlo simulation
ML	machine learning
ISSA-SVR	improved sparrow search algorithm and the support vector regression
BPNN	back-propagation neural network
ISCA	improved sine–cosine algorithm
SVM	support vector machine
CR	polychloroprene rubber
AN2 and AN5	cylindrical hourglass-shaped specimens with notch radii equal to 1.75 and 4.75 mm, respectively.
AE2 and AE42	axisymmetric specimens with curvature radii equal to 2 and 42 mm, respectively.
DENT	double edge notched tension specimen
CCT	center cracked tension specimen
PS	pure shear or planar tension specimen with or without a precut
phr	parts per hundred rubber
NBR	nitrile butadine rubber
CIP	carbonyl iron powder/particle
